# Quantitation of phosphohistidine in proteins in a mammalian cell line by ^31^P NMR

**DOI:** 10.1371/journal.pone.0273797

**Published:** 2022-09-01

**Authors:** Mehul V. Makwana, Mike P. Williamson, Richard F. W. Jackson, Richmond Muimo

**Affiliations:** 1 Department of Chemistry, The University of Sheffield, Sheffield, United Kingdom; 2 Department of Infection, Immunity and Cardiovascular Disease, The University of Sheffield, Sheffield, United Kingdom; 3 School of Biosciences, The University of Sheffield, Sheffield, United Kingdom; George Washington University, UNITED STATES

## Abstract

There is growing evidence to suggest that phosphohistidines are present at significant levels in mammalian cells and play a part in regulating cellular activity, in particular signaling pathways related to cancer. Because of the chemical instability of phosphohistidine at neutral or acid pH, it remains unclear how much phosphohistidine is present in cells. Here we describe a protocol for extracting proteins from mammalian cells in a way that avoids loss of covalent phosphates from proteins, and use it to measure phosphohistidine concentrations in human bronchial epithelial cell (16HBE14o-) lysate using ^31^P NMR spectroscopic analysis. Phosphohistidine is determined on average to be approximately one third as abundant as phosphoserine and phosphothreonine combined (and thus roughly 15 times more abundant than phosphotyrosine). The amount of phosphohistidine, and phosphoserine/phosphothreonine per gram of protein from a cell lysate was determined to be 23 μmol/g and 68 μmol/g respectively. The amount of phosphohistidine, and phosphoserine/phosphothreonine per cell was determined to be 1.8 fmol/cell, and 5.8 fmol/cell respectively. Phosphorylation is largely at the N3 (*tele*) position. Typical tryptic digest conditions result in loss of most of the phosphohistidine present, which may explain why the amounts reported here are greater than is generally seen using mass spectroscopy assays. The results further strengthen the case for a functional role of phosphohistidine in eukaryotic cells.

## Introduction

The reversible covalent post-translational modification (PTM) of proteins by phosphorylation is one of the most common and important ways of regulating protein function in eukaryotes [1]. The most familiar phosphorylation sites are serine, threonine and tyrosine, yielding O-linked phosphates, which are kinetically stable under normal cellular conditions. The relative levels of phosphorylation at serine, threonine and tyrosine (pSer, pThr and pTyr) in unstimulated cells are approximately 24.6: 4.3: 1 [2]. Despite the relatively low amounts of pTyr in cells, phosphorylation of tyrosine is important because it often initiates signaling pathways. Defects in tyrosine phosphorylation therefore are often linked to disease, in particular to cancers. Unsurprisingly, there have been a very large number of studies on phosphorylation at serine, threonine and tyrosine [3].

By contrast, phosphorylation at the “non-canonical” residues histidine, arginine, lysine, aspartate, glutamate and cysteine has been much less studied [4–7]. These PTMs are less chemically stable and have short lifetimes in cells, which makes them harder to study [8]. Because of their lability, they are under-represented in databases [1,9] and poorly understood.

Of these non-canonical phosphorylations, phosphohistidine (pHis) is probably the most important. It forms a critical part of two-component signaling systems in bacteria [10], which are also used by lower eukaryotes and plants, but not by higher eukaryotes. It was first observed in eukaryotes in 1962 [11], and has attracted attention recently stimulated by the finding that the enzyme histidine phosphatase, LHPP, has a role as a tumor suppressor [12]. Since this observation, LHPP has been found to play a role in a wide range of cancers [13], with the suggestion that its function is to inhibit the PI3 kinase / protein kinase B (AKT) signaling pathway [14]. Several eukaryotic enzymes have now been identified that phosphorylate histidine, or dephosphorylate pHis [15], and there is a database of histidine phosphorylation sites [16]. A mass spectroscopy study in 2019 identified a large number of pHis sites, and concluded that the amount of noncanonical phosphorylation was approximately the same amount as pTyr: Hardman et al [2] estimated ratios of pTyr: pHis: pAsp: pGlu: pLys: pArg of 1: 0.6: 1: 0.8: 0.8: 0.9.

Here we report an NMR-based method for analyzing pHis content, which avoids neutral and acidic pH, and we conclude that the amount of pHis in the cell is considerably greater than previously estimated, reaching as much as one third of the total amount of pSer and pThr. pHis is unique among amino acid phosphoryl PTMs in that the phosphate can be attached at either N1 (the π or *pros* position) or N3 (the τ or *tele* position). We show that the majority of substitution is at the N3 position. This is not surprising as it is expected to be the more stable phosphorylation site [17].

## Results

### NMR characterisation of synthetic pHis

3-(τ)-pHis and 1-(π)-pHis were synthesized by the reaction of His with potassium phosphoramidate in water, followed by purification, to allow determination of reference ^31^P chemical shifts of each isomer [18]. Chemical shifts of—4.99 ppm and—5.76 ppm were observed for 3-pHis and 1-pHis respectively (S1 Fig), entirely consistent with values previously reported [18,19].

### Chemically phosphorylated myoglobin

**Fig 1:**
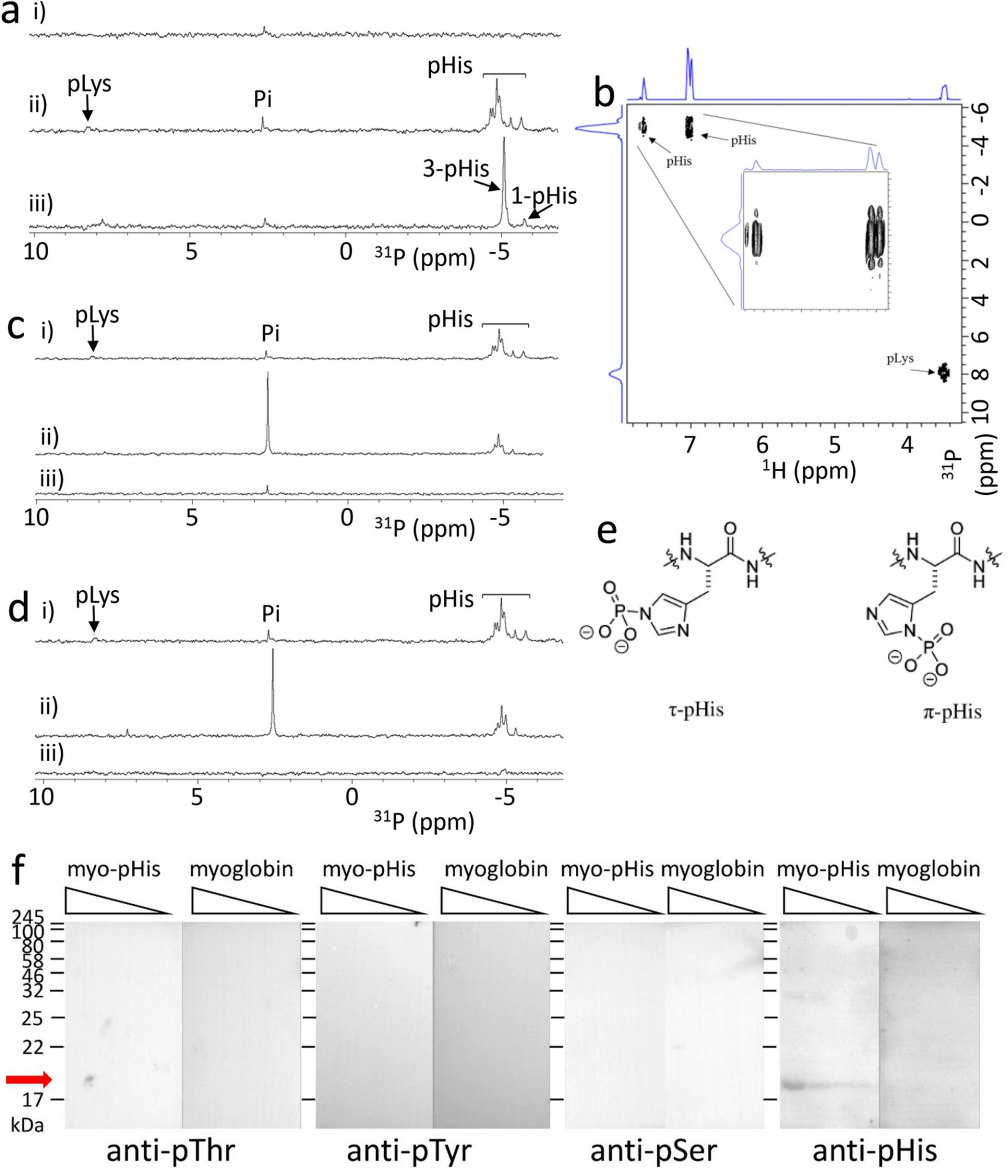
^31^P NMR spectra of Myo-pHis. a) Phosphorylation of histidines in myoglobin i) Native myoglobin, pH 10.8; ii) Myo-pHis, pH 10.8, following phosphorylation with potassium phosphoramidate, buffer exchange to pH 10.8, and concentration (18.2 mg/mL); iii) Denatured myo-pHis. The Myo-pHis sample used was the same sample as Fig 1aii. The signals from 3-pHis and 1-pHis are indicated. This distinction is not possible in the native protein because of the spread of chemical shifts due to the folded structure. b) HMBC NMR spectrum of Myo-pHis, sample from Fig 1aiii. The expansion inset shows the slightly different ^31^P chemical shifts for the 3-pHis and 1-pHis; c) Effect of acidic conditions i) Myo-pHis, pH 10.8, prepared as Fig 1aii; ii) Myo-pHis after treatment with trypsin, at 37°C for 16 h at pH 8.0. iii) Myo-pHis at pH 10.8 after trypsinization and subsequent desalting; d) Effect of trypsinisation i) Myo-pHis at pH 10.8, prepared as Fig 1aii; ii) Myo-pHis after reduction with DTT, alkylation with IAA and subsequent trypsinisation at pH 8.0. iii) Myo-pHis at pH 10.8, after trypsinization and desalting. e) Structures of τ-(N3)-pHis and π-(N1)-pHis. f) Western blots of myoglobin-pHis and myoglobin probed with phosphoantibodies. From left to right: pHis antibody (ab231709); pTyr antibody (pY20; pSer antibody (Q5); pThr antibody (Q4). For each blot, the gel has four lanes, with decreasing amounts of protein: 1, 0.5, 0.25, and 0.125 mg. The red arrow marks the expected position of myoglobin.

Mass spectroscopy (MS) studies have shown that myoglobin can be phosphorylated selectively on His residues using potassium phosphoramidate in water to give myoglobin-pHis (Myo-pHis) [2,20]. We therefore decided to use Myo-pHis as a standard to test suitable conditions for NMR measurements of cellular proteins. Potassium phosphoramidate was used to phosphorylate myoglobin in water, at 25°C for 15 h [20]. pHis is relatively stable under basic conditions (pH 10–12), and unstable under neutral and acidic conditions [17], and therefore Myo-pHis was buffer exchanged into 0.1 M Na_2_CO_3_/NaHCO_3_, at pH 10.8 (which also removed any unreacted potassium phosphoramidate), and subsequently concentrated and analyzed by ^31^P NMR spectroscopy. Multiple pHis residue signals were observed in the chemical shift range of—4.40 to—5.76 ppm (Fig 1A); additionally we observed an inorganic phosphate (Pi) signal at 2.57 ppm and a signal at 8.27 ppm which is discussed below and shown to derive from pLys [21] using an HMBC experiment (Fig 1B).

To reduce the tertiary structural effect of myoglobin on the pHis chemical shifts, Myo-pHis was denatured with 7 M urea (Fig 1aiii). Two distinct sets of signals in the ranges—4.80 to—5.38 ppm, and—5.52 to—5.98 ppm were observed in the ^31^P NMR spectrum of the denatured Myo-pHis. In heteronuclear multiple bond correlation (HMBC) NMR experiments of denatured Myo-pHis, cross peaks between ^1^H NMR aromatic protons (with chemical shifts of 6.97, 7.06 and 7.59 ppm) and ^31^P NMR pHis residue signals are observed, which arise from 3-bond correlations between the ^31^P and two aromatic protons of 3-pHis, plus one aromatic proton of 1-pHis (Fig 1R), confirming phosphorylation had indeed occurred on His residues (Fig 1B). This spectrum also confirms the assignment of the pLys signal, from the correlation between the pLys ^31^P (+8 ppm) and pLys Hε (3.48 ppm). On Western blots of Myo-pHis against commercially available pHis, pSer, pThr, and pTyr antibodies (Fig 1F), low levels of pSer, pThr, and pTyr residues were detected, which is in agreement with the ^31^P NMR spectrum (Fig 1) and reported MS data for Myo-pHis [2,20]. Further confirmation of specific phosphorylation at histidine is that after acid treatment (~ pH 4) and heating (90°C) of Myo-pHis, all proposed pHis and pLys residue signals were abolished, giving rise to an increased Pi signal suggesting the presence of acid labile residues (S2 Fig).

pHis isomer distinction in a folded protein or peptide cannot always be made by comparing the pHis residue ^31^P NMR chemical shifts with pHis amino acid standards [22]. However, comparison of the denatured Myo-pHis chemical shift regions of ~ - 4.94 ppm (major) and ~ - 5.61 ppm (minor) with the chemical shifts of synthetic 3-pHis and 1-pHis amino acid standards (S1 Fig) suggests that the major pHis isomer in Myo-pHis was 3-pHis. 3-pHis is the more stable isomer of pHis [17] and under conditions used to phosphorylate His, 3-pHis would be expected to be the major isomer present in Myo-pHis. No peaks attributable to phosphorylation at both the 1- and 3-positions could be observed.

To determine the number of pHis residues in the sample, a capillary tube containing an external standard (triphenylphosphine oxide, 10 mol % Cr(acac)_3_ in CDCl_3_) was used as an insert into the NMR tube containing Myo-pHis. Triphenylphosphine oxide was chosen as the external standard because it is a stable compound with a chemical shift ~ 30 ppm away from the pHis chemical shift region. The average number of pHis residues per myoglobin protein was determined to be 5.6 and the amount of pHis residues per gram of myoglobin was determined to be 0.32 mmol/g (S1 File). There are eleven His residues in myoglobin, all of which have been observed by MS to be phosphorylated by potassium phosphoramidate [20], and the quantitation here would suggest that on average approximately half of the His residues are phosphorylated. Potassium phosphoramidate can phosphorylate both His imidazole nitrogens, and a small degree of double phosphorylation cannot be excluded for Myo-pHis [18].

Trypsinization and desalting are typical steps used to process proteins from a cell lysate before MS analysis, and the conditions used are likely to result in the destruction of some pHis. To assess the impact of trypsinization conditions, Myo-pHis was trypsinized using typical conditions (37°C, pH 8.0, 16 h) [23] and then analyzed by ^31^P NMR spectroscopy (Fig 1C). There was a significant change in the integration ratio of Pi and pHis residue signals from 1:15.8 for Myo-pHis (Fig 1ci), to 1:0.97 for trypsinised Myo-pHis (Fig 1cii). In addition, pLys residue signals also decreased. These results show extensive hydrolysis of both pHis and pLys residues under typical trypsinization conditions. The trypsin-treated Myo-pHis (Fig 1cii) was subsequently desalted using C-18 resin following typical conditions, which are more acidic. In the ^31^P NMR spectrum of the desalted sample, no pHis residue signals were observed despite protein being still present, suggesting desalting also has a destructive effect on pHis residues (Fig 1ciii) [24]. Hardman *et al*. have detected Myo-pHis peptides by MS using milder trypsinization: Myo-pHis was first treated with dithiothreitol, and then iodoacetamide before the addition of trypsin at 30°C, pH 8.0, 16 h followed by desalting at neutral pH [2]. Under these milder trypsinization conditions, the integration ratios of Pi and pHis signals in the ^31^P NMR spectra (Fig 1di and 1dii) changed from 1:15.8 for Myo-pHis to 1:1.35 for trypsinized Myo-pHis showing a modest reduction in the amount of pHis residues that were hydrolyzed compared to typical trypsinization condition (*vide supra*). Following Hardman *et al*., Myo-pHis treated with trypsin was desalted using a C-18 resin and neutral solutions. The sample was subsequently analyzed by ^31^P NMR spectroscopy (Fig 1diii). Relatively small pHis signals were observed when compared to the spectrum of Myo-pHis treated with trypsin (Fig 1dii). The ^31^P NMR data thus shows that even using milder trypsinization and desalting conditions there was a significant loss of pHis. We therefore propose that the amount of pHis detected by Hardman *et al*. is an underestimate of the true amount.

### Quantitation of pHis in mammalian cells

**Fig 2:**
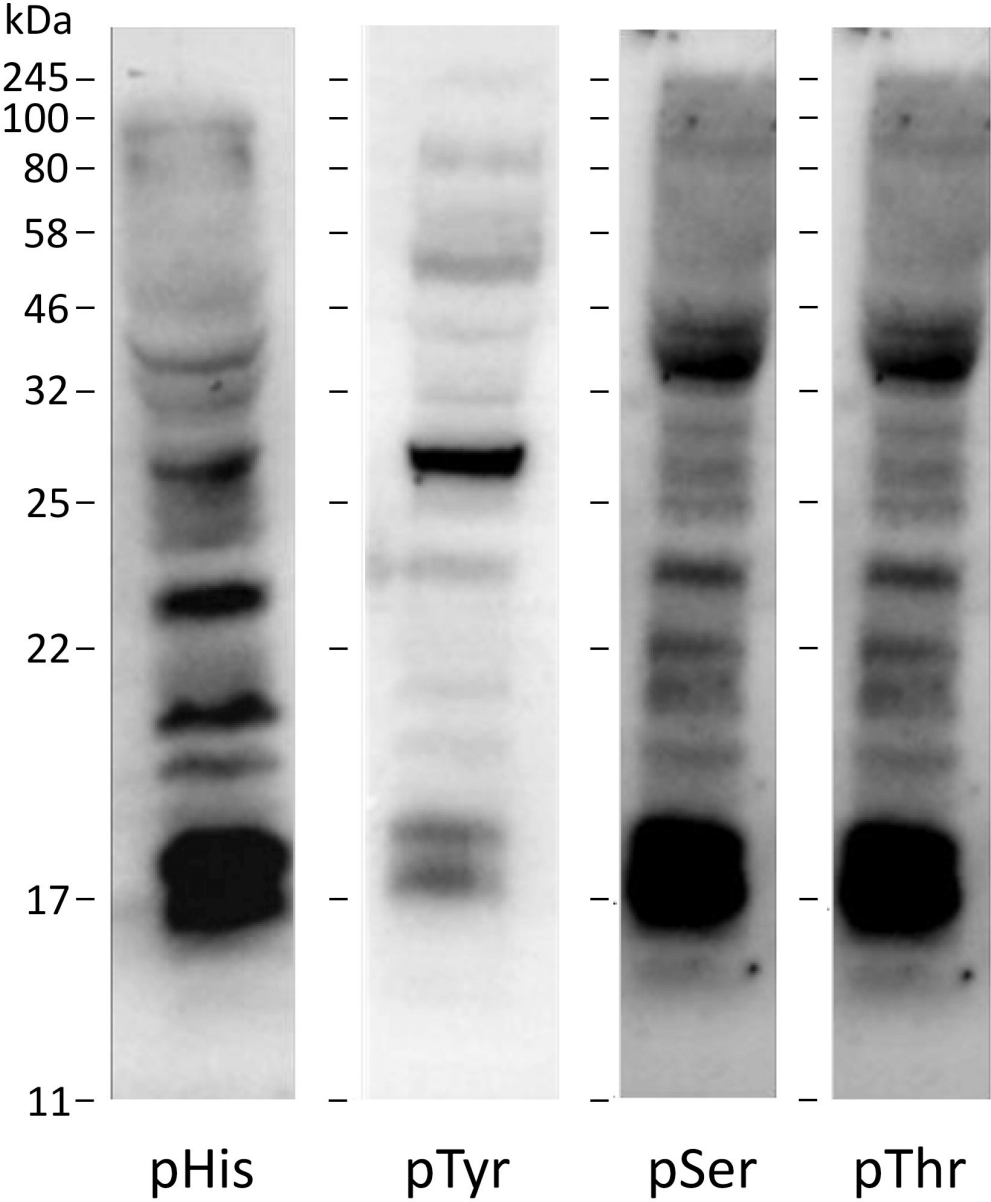
Western blots of 16HBE14o- cell lysates probed with phosphoantibodies. 16HBE14o- cell lysate (100 μg of protein) was probed with pHis (ab2317090), pTyr (pY99), pThr, or pSer (Q5) antibody.

The quantitative ^31^P NMR spectroscopic analysis of pHis residues developed on Myo-pHis was then applied to a more complex sample, namely the proteins from a cell lysate. Airway epithelia are known to produce His phosphorylated proteins and we therefore used the 16HBE14o- cell line, which is a well-known airway epithelial cell line [25,26]. A Western blot of 16HBE14o- cell lysate using a pHis antibody (ab2317090) detected several pHis bands confirming the presence of pHis residues (Fig 2). Western blot and cell lysis procedures used pHis stabilizing conditions (see Methods). The pHis antibody selectivity was validated by acid treatment of the 16HBE14o- cell lysate which reduced the pHis signal, while base treatment retained the pHis signal (S3 Fig). Similarly, Western blots of 16HBE14o- using pTyr, pThr, and pSer antibodies confirmed the presence of pTyr, pThr and pSer residues (Fig 2).

To analyze the ^31^P NMR signals of phosphoproteins from 16HBE14o- cell lysates, it was important to use a procedure that removed other phosphorus containing molecules (e.g. nucleoside triphosphates, oligonucleotides etc.) and as much DNA/RNA as possible, which could otherwise potentially dominate the ^31^P NMR spectrum. It was also important to keep the solution alkaline at all times, because pHis is degraded rapidly in acidic or neutral conditions. The preferred buffer was a weak base (such as Na_2_CO_3_/NaHCO_3_, pH 10.8, *vide supra*) because pSer and pThr are known to decompose by β-elimination on overnight treatment with a strong base such as 1 M NaOH, 37°C [4,27]. Under these conditions, both pSer and pThr were found to be stable for more than two days (S4 Fig). Therefore, a robust, rapid and simple procedure was developed for preserving the native phosphorylation states of all phosphorylated amino acids.

**Fig 3:**
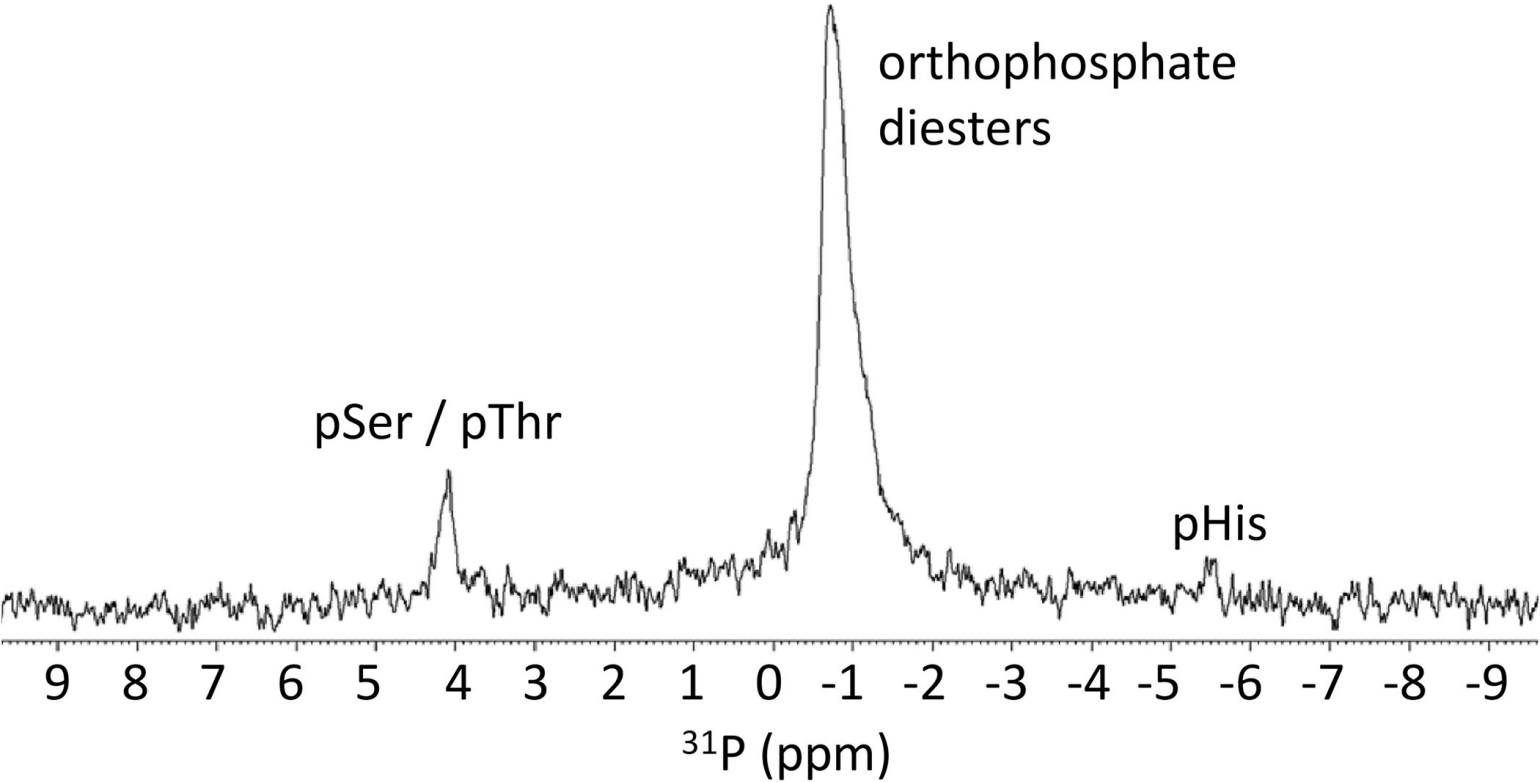
^31^P NMR spectrum of proteins from the 16HBE14o- cell lysate in Na_2_CO_3_/NaHCO_3_, 7 M urea, 10% (v/v) D_2_O, pH 10.8. The orthophosphate diester peak is assumed to contain DNA, RNA and phospholipids.

Lysis buffers containing urea as denaturant have been used to prepare cell lysates for MS analysis of pHis [2,28,29]. Thus, 16HBE14o- cells were lysed on culture plates using 0.1 M Na_2_CO_3_/NaHCO_3_, 7 M urea, pH 10.8 and were immediately subjected to sonication. Sonication breaks DNA into small pieces which are subsequently removed along with other phosphorus containing small molecules during buffer exchange (S5 Fig), while the denaturant ensures that there is reduced or no enzymatic activity; in particular that the activity of phosphatases and phosphoryl transferases are kept to a minimum. Using this procedure, pHis and pSer/pThr signals [30,31] in the ^31^P NMR spectrum were reproducibly observed (Fig 3). Additional signals from -0.05 to -1.70 ppm are also observed in Fig 3. The chemical shift regions in which these signals are present are characteristic of orthophosphate diesters [32], and thus have been assigned accordingly. In the basal state, the expected abundance of pTyr residues in 16HBE14o- cells is low [33] and observable ^31^P NMR pTyr residue signals were not expected. Under the conditions used, pTyr is stable [34] and is expected to have a chemical shift around 0.29 ppm (S1e Fig), but could not be observed because any pTyr residue signals would overlap with signals due to orthophosphates. The pHis signals have an integrated intensity approximately 30% that of the combined pSer/pThr signals, and any pLys signals were of too low intensity to be observed (Fig 3). The chemical shifts of the pHis signals suggest that the majority of the pHis are present as 3-pHis, with a small amount of 1-pHis (see also S5 Fig).

An alternative DNA removal strategy described by Antonioli *et al*. uses basic conditions to extract DNA, but precipitates the cell lysate proteins rather than keeping them in solution [35]. Application of this method to the 16HBE14o- cell lysate (lysed in 30 mM Tris-HCl, 50 mM NaCl, complete protease inhibitor, pH 9.0) and subsequent ^31^P NMR spectroscopic analysis showed signals characteristic of orthophosphate diesters and pSer/pThr residues but no signals characteristic of pHis residues (S6 Fig). Protein precipitation conditions which have been previously used in the analysis of pHis by MS [29] gave similar results. Thus, it appears that protein precipitation is detrimental in the analysis of pHis proteins from a cell lysate by ^31^P NMR spectroscopy.

For additional validation, the proteins from the 16HBE14o- cell lysate analyzed in S6 Fig were chemically phosphorylated with potassium phosphoramidate which gave rise to ^31^P NMR signals from—4.68 to—6.13 ppm (S7 Fig), confirming that the signals observed in Fig 3 are indeed pHis residues. Furthermore, as observed with Myo-pHis, acid treatment (~ pH 4) and heating (90°C) of the proteins from the 16HBE14o- cell lysate resulted in loss of the pHis residue signals whilst giving rise to a Pi signal (S8 Fig).

**Fig 4:**
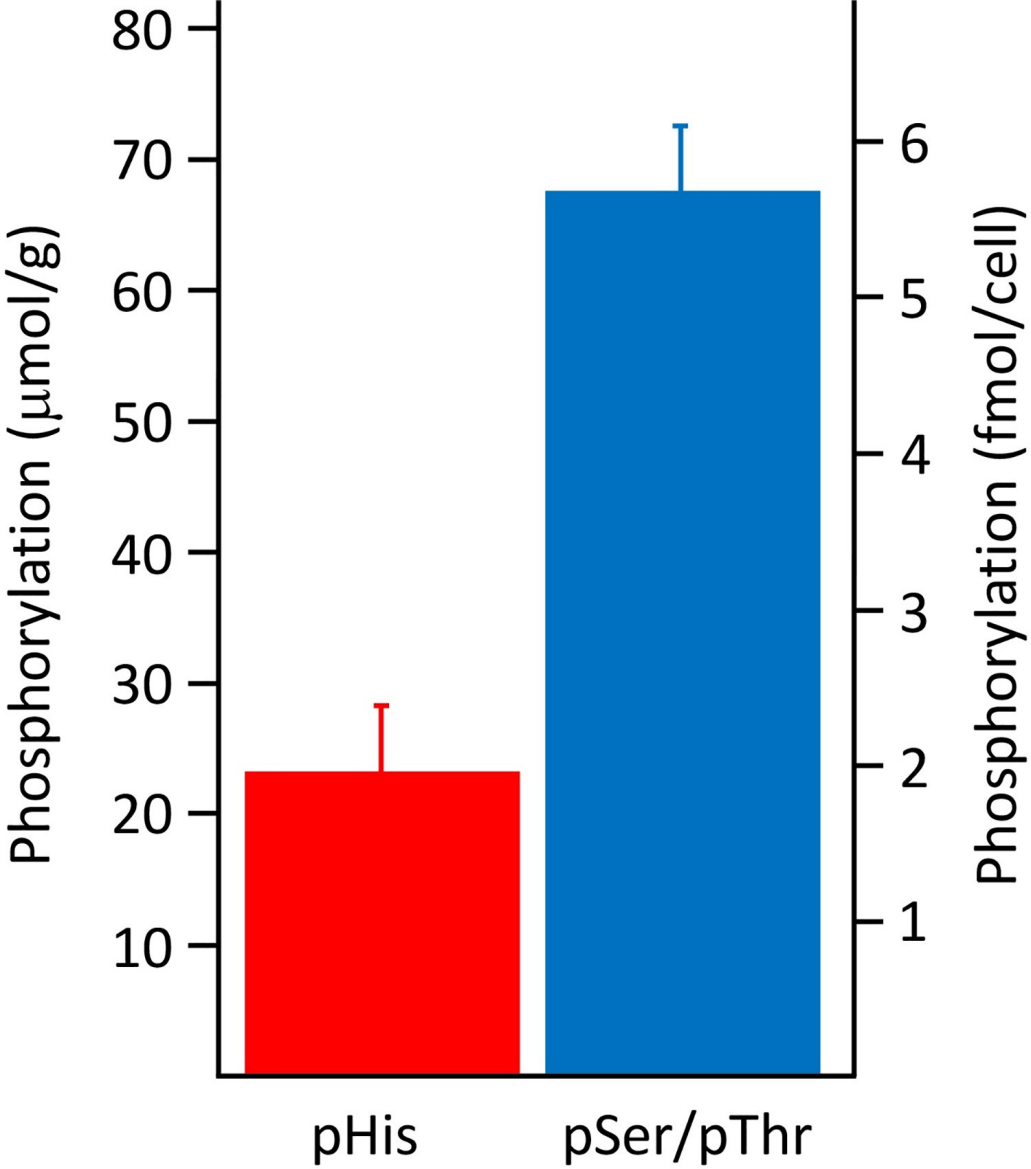
Quantification of pHis and pSer/pThr in 16HBE14o- cells using ^31^P NMR spectroscopy. Error bars show the standard error of the mean (*n* = 3).

Previously, relative phosphorylated amino acid abundances have been reported for proteins from a cell sample either as a percentage or a ratio [36]. However, since absolute quantitation is possible with ^31^P NMR spectroscopy, it is possible to determine the absolute amounts of different phosphorylated amino acid residues, as well as the relative amounts. In a triplicate ^31^P NMR spectroscopy analysis, the pHis: pSer/pThr residue signal integration ratio was found to be on average 0.34: 1 (S9 and S10 Figs). Using an external standard, the average amounts of pHis, and pSer/pThr residues per mass of protein cell lysate were determined to be 23 μmol/g, and 68 μmol/g respectively (S1 Table). The amounts of pHis, and pSer/Thr residues per cell were determined to be 1.8 fmol/cell, and 5.8 fmol/cell respectively (Fig 4; S2 Table).

**Fig 5:**
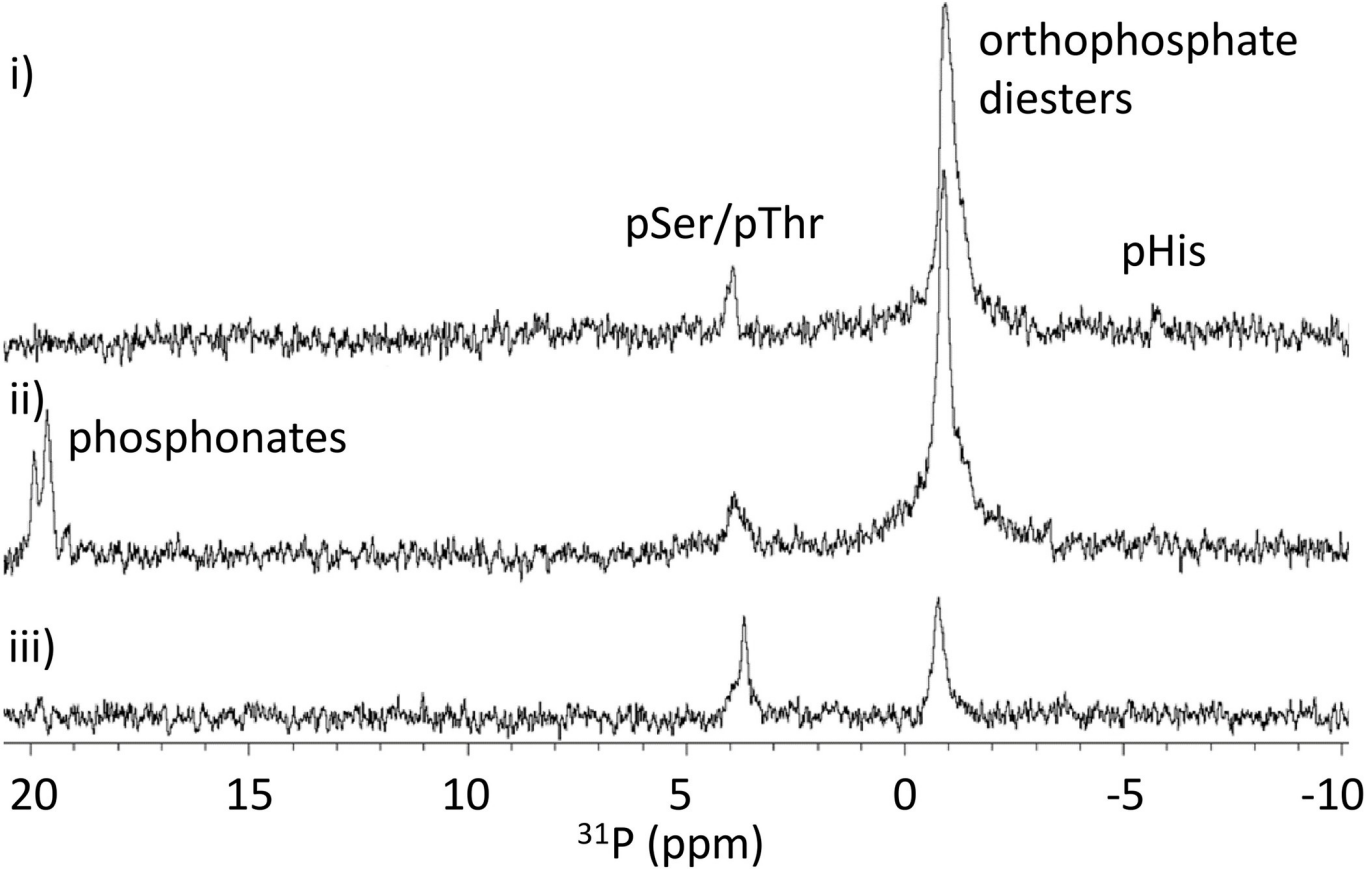
^31^P NMR spectra of proteins from 16HBE14o- cell lysate after trypsinization and desalting. **i**) Proteins from the 16HBE14o- cell lysate at pH 10.8 prepared as Fig 3; **ii**) Same sample after buffer exchange and treatment with trypsin, at 37°C for 16 h at pH 8.0; **iii**) Same sample after desalting. Signal assignments have been made using literature chemical shift values [30–32,40].

As observed with the Myo-pHis standard, the intensities of the ^31^P NMR signals from the 16HBE14o- cell lysate assigned to pHis residues were found to reduce upon trypsinization (Fig 5). However, unlike Myo-pHis, new signals from 19.06 to 20.38 ppm (corresponding to the phosphonate region of the spectrum) in the ^31^P NMR spectrum of trypsinized proteins from the 16HBE14o- cell lysate were observed, but no Pi signal. Phosphonates contain a phosphorus-carbon bond and such bonds are likely to arise from the reaction of a nucleophilic carbon species and phosphate electrophile. The mechanism by which these phosphonates arise and their identity is unclear but previous studies have observed phosphonate lipids giving similar chemical shift values [37]. Since high urea concentration reduces trypsin activity [23], the 16HBE14o- cell lysates were buffer exchanged in preparation for trypsinization. It is likely that during this step, enzymatic activity in the 16HBE14o- cell lysates was partially restored. Whether the emergence of the phosphonate signals and absence of Pi signal was due to a chemical or enzymatic transfer of the phosphoryl group is again unclear. In-solution trypsinization for MS sample preparations generally uses cell lysates that have not been dialyzed but have undergone reduction and alkylation under denaturing conditions, after which the denaturant is diluted, and the sample is treated with trypsin [2,38]. For simplicity, and to minimize sample handling, reduction and alkylation was not carried out here. After desalting, the phosphonate signals were absent. Phosphonates are generally chemically stable, and we therefore conclude that the phosphonates are low molecular weight lipid phosphonates that were removed by the dialysis. The presence of the orthophosphate diester signals after the desalt suggests that a significant fraction of these orthophosphate diesters are covalently linked to proteins, which is not uncommon [39]. For comparison purposes, proteins from the 16HBE14o- cell lysate were also trypsinized and desalted following conditions by Hardman *et al*. [2], which included reduction and alkylation steps. A ^31^P NMR spectrum similar to Fig 5iii was observed (S11 Fig).

## Discussion

This study demonstrates unexpectedly high amounts of pHis in mammalian 16HBE14o- cells. This may be due to its labile nature under acidic and near neutral pH experimental conditions, which may have reduced the levels of pHis seen in earlier mass spectroscopy based studies. By using a pHis stabilizing denaturing lysis buffer, and minimal sample processing steps followed by a nondestructive analytical technique (i.e. ^31^P NMR spectroscopy), the amount of pHis was determined to be on average approximately one third the combined amount of pSer/pThr in proteins from the 16HBE14o- cell lysate. It has been shown that the relative abundance of pSer, pThr and pTyr is approximately 55:10:1 in human cells [41], or alternatively 24.6:4.3:1 [2], implying that pHis is likely to be about 10–20 times more abundant in human cells than pTyr, consistent with the results shown here.

The study demonstrates that ^31^P NMR spectroscopy can be applied to the absolute quantitation of pHis (and other phosphoamino acids) in a mammalian cell. The study also shows that ^31^P NMR spectroscopy can be used as a tool to monitor changes in phosphorylation resulting from subjecting a sample to different conditions. The simplicity of the procedure (only three sample processing steps before spectroscopic analysis: lysis, sonication, and simultaneous buffer exchange/concentration) highlights the potential broad applicability of the method in phosphoamino acid residue analyses of various cells/tissues or even small multicellular organisms. Since absolute quantitation is possible with ^31^P NMR spectroscopy, phosphorylation can be expressed in moles per gram of protein cell lysate or per cell, thereby allowing direct comparison between different cells or cell types.

Importantly, we show here that subjecting pHis-containing samples to typical mild trypsinization and desalting conditions used previously in MS studies results in loss of a significant fraction of pHis (Figs 1 and 5). Our results suggest that even gentler conditions are required for reliable quantitation, as developed here.

Using a pHis stabilizing denaturing lysis buffer, the ^31^P NMR spectrum in Fig 3 shows that, in the basal state, pHis residues are more abundant in mammalian 16HBE14o- cells than previously thought. Until now Ser, Thr and Tyr phosphorylated amino acid residues in mammalian cells have been thought to be the major players in cell signaling in eukaryotic cells. However, this study suggests that pHis may also play an important role in cell signaling in higher eukaryotes, in addition to its established role in prokaryotes and lower eukaryotes [4,42]. One example of the importance of pHis is illustrated by the need to relay nascent ATP away from the outer rim of the mitochondrion to avoid negative charge build up such that energy production is compromised if phosphorylated histidine is unavailable [43]. For example, mutation of NME3, a member of the nucleoside diphosphate kinase (NDPK) family with activity as a histidine kinase, underlies a fatal mitochondrial disorder [44], and double deletion of murine NME1 and NME2 is incompatible with postnatal life [45].

The importance of pHis to higher eukaryotes is further illustrated by the growing number of histidine kinases, phosphotransferases [46] and phosphatases that have been characterised. This includes the NME family of likely histidine kinases [47,48], as well as the protein histidine phosphatase LHPP which has multifaceted activity as a tumour suppressor for a range of cancers [12,14], and the histidine phosphatase PHPT1, which is an oncogene involved in lung cancer [49]. To date there is good evidence for these proteins’ involvement in cancers, but little biochemical evidence that their function is due to histidine phosphorylation/dephosphorylation. The remarkably high incidence of pHis revealed here makes this a likelihood.

We note a report that the metastasis suppressor Mn23-H1 can transfer phosphate from histidine to a serine on the kinase suppressor of Ras (KSR), a scaffold protein for the mitogen-activated kinase cascade [50]. This is an interesting regulatory function for pHis; it also emphasises the importance of denaturing conditions during analysis of the phosphoproteome, to avoid artifactual intermolecular transfer of phosphate before analysis.

In conclusion, this study introduces a ^31^P NMR spectroscopy method for absolute quantification of pHis and pSer/pThr in mammalian cells, thereby in principle allowing direct comparison between different cell types. This simple, direct method has allowed a longstanding question about the likely importance of pHis in mammalian cell biology to be addressed by demonstrating that pHis is a relatively abundant phosphorylation PTM in 16HBE14o- cells under basal conditions.

## Methods

### NMR spectroscopy

Norell® Standard Series™ 5 mm NMR tubes were used. ^31^P spectra were acquired on a Bruker AVANCE III or Bruker AVANCE III HD spectrometer at 162.0 MHz or 202.5 MHz respectively. Data were acquired using an acquisition window of 9.69 kHz or 12.1 kHz respectively (59.9 ppm) with 32 k acquisition points (14–15 hours acquisition time), a 30° pulse and a relaxation delay of 8 s. For all critical measurements, the measurement was repeated a second time to check for possible loss of phosphate during signal acquisition, and none was seen. This pulse angle and recycle delay combination had previously been determined to be sufficiently long for complete relaxation, thus quantitative for all ^31^P containing species in the samples, including those with external standard which was doped with chromium(III) acetylacetonate to aid relaxation. The standard was contained in Norell® high throughput 3 mm NMR sampling tubes. The capillary contained 2 or 4 mM triphenylphosphine oxide, 10 mol % chromium(III) acetylacetonate, dissolved in 150 μL of deuterated chloroform (CDCl_3_). ^1^H-^31^P HMBC spectra were acquired on a Bruker AVANCE III 400 spectrometer, using 1664 scans for each of 128 increments over an acquisition window of 9.7 kHz and 3.6 kHz (2 k points) in F1 and F2 respectively and optimized for a long-range coupling constant of 10 Hz [19,21]. pSer, pThr, pTyr, 3-pHis, 1-pHis, and inorganic phosphate NMR spectra were recorded at room temperature. ^1^H NMR spectra were recorded using a Bruker AVANCE 400 spectrometer operating at 400.13 MHz, or Bruker AVANCE III HD 400 spectrometer operating at 400.23 MHz. ^13^C NMR spectra were recorded using a Bruker AVANCE 400 spectrometer operating at 100.61 MHz, or Bruker AVANCE III HD 400 spectrometer operating at 100.64 MHz. ^31^P NMR spectra were recorded using a Bruker AVANCE III HD 400 spectrometer operating at 162.02 MHz. Chemical shifts were measured relative to the residual solvent and expressed in parts per million (δ). The multiplicities are defined as s = singlet, d = doublet, t = triplet, q = quartet, quint. = quintet, sex. = sextet, m = multiplet, br = broad.

### Materials and general experimental methods

All reagents used were purchased from Fluorochem, Millipore, Sigma, Alpha Aeser, VWR International, Thermo Fisher Scientific, Qiagen, and Bio-Rad Laboratories, Inc. Ultrapure water (18 MΩ) was used to make buffers and solutions unless stated otherwise. All solvents were of HPLC grade. ULTRA PURE AccuGel^TM^ 29:1, 30 (w/v) 29:1 acrylamide: bis-acrylamide solution (gas stabilised) was used to make SDS-PAGE gels. Phosphate buffered saline (PBS): 1.8 mM monopotassium phosphate, 10 mM disodium phosphate, 2.7 mM potassium chloride, 137 mM sodium chloride, pH 7.4. Pierce^TM^ BCA protein assay kit, and Bio-Rad Bradford protein assay was used to estimate proteins. Thermo Scientific SuperSignal^TM^ West Pico PLUS chemiluminescent substrate was used to develop blotted membranes. Sigma-Aldrich Medium 199 with Earle′s salts, sodium bicarbonate, phenol red, without L-glutamine was used to culture 16HBE14o-. Gibco low endotoxin heat-inactivated fetal bovine serum was used to supplement media. Sigma-Aldrich Penicillin-Streptomycin; 10,000 units penicillin/mL and 10 mg streptomycin/mL was used to supplement media. Sigma-Aldrich 200 mM L-Glutamine solution was used to supplement media. Sigma-Aldrich trypsin from bovine pancreas was used to trypsinize proteins. Sigma Trypsin-EDTA solution (1 ×, sterile; sterile-filtered, BioReagent, suitable for cell culture, 0.5 g porcine trypsin and 0.2 g EDTA • 4Na per liter of Hanks′ Balanced Salt Solution with phenol red) was used to trypsinize adhered 16HBE14o- cells. Roche complete protease inhibitor cocktail, Sigma-Aldrich myoglobin from equine heart and gelatin from cold water fish skin, New England Biolabs pre-stained protein standard, broad Range (11–190 kDa), and Abcam pHis (ab2317090) at 1/1000 (v/v) dilution, Invitrogen pTyr (pY99) at 1/2000 (v/v) dilution, Qiagen pThr (Q7) at 1/200-1/2000 (v/v) dilution, and Qiagen pSer (Q5) at 1/200-1/2000 dilution were used. Dako rabbit anti-goat at 1/2000 (v/v) dilution, Dako mouse anti-rabbit at 1/2000 (v/v) dilution, and Abcam goat anti-Mouse IgG H&L (ab97023) at 1/10000 (v/v) dilution, immunoglobulins/ horseradish peroxidase were used as the secondary antibodies. Millipore® PVDF membranes (0.45 μm) were used to blot proteins. Silica gel 40–60 μm from VWR International was used. Merck TLC Silica gel 60 F254 TLC plates were used, and compounds were visualized by UV light (254 nm), 5% (wt/v) ninhydrin in methanol. Sartorius Vivaspin 20 (3 and 10 kDa MWCO) were used for dialysis and concentration of protein solutions. Western blotted membranes were visualised using a Bio-Rad ChemiDoc^TM^ XRS+ with image Lab^TM^ software. Infrared spectra were recorded using a Perkin Elmer Paragon 100 FTIR Spectrophotometer by attenuated total reflectance (ATR). Only selected peaks are reported, and the absorption maxima are given to the nearest wavenumber (cm^-1^). High-resolution mass spectra were measured using an Agilent Technologies 653 Accurate-Mass Q-TOF LC/MS operating in electrospray mode.

#### Synthesis of amino(potassiooxy)phosphinic acid (Potassium phosphoramidate)

To a stirred mixture of water (100 mL), and 32% (wt/wt) ammonia (50 mL), ice cold phosphorus(V) oxychloride (15.1 g, 9.2 mL, 98.5 mmol, 1.0 equiv.) was added dropwise at 4°C, over 25 minutes. Effervescence was observed and the solution was stirred for a further 15 minutes. Acetone (500 mL) was added and the mixture was vigorously stirred for 5 minutes. The aqueous layer was partitioned and acidified with glacial acetic acid to pH 6. A precipitate was observed. After keeping the suspension at -20°C for 60 minutes, the precipitate was filtered off and washed sequentially with ethanol (20 mL) and diethyl ether (20 mL). The air-dried crystals were added in small portions to 50% (wt/v) potassium hydroxide (23 mL), and then heated to 60°C for 20 minutes. The mixture was allowed to cool to room temperature and then acidified with glacial acetic acid to pH 6. The suspension was poured into ethanol (1150 mL) and left to stand at room temperature for 60 minutes. The precipitate was collected on a sintered funnel and washed sequentially with ethanol (2 x 30 mL) and diethyl ether (2 x 30 mL) and dried under reduced pressure. Potassium phosphoramidate (6.21 g, 47%) white powder: ν_max_(thin film)/cm^-1^ 2849, 2473, 2175, 1614, 1462, 1413, 1150, 1073; ^31^P NMR (162 MHz, D_2_O) -3.33. The characterization data are comparable to the literature [51].

#### Synthesis of 2-amino-3-(3-phosphonoimidazol-4-yl)propanoic acid (1-(π)-pHis)

L-Histidine (0.251 g, 1.62 mmol, 1.0 equiv.), potassium phosphoramidate (0.57 g, 4.21 mmol, 2.6 equiv.) and water (4.5 mL) were stirred at 25° C for 40 minutes. 1-pHis was chromatographically purified as described below. The combined product fractions were concentrated by rotary evaporation at 25°C to approximately 4 mL whilst maintaining a pH between 10–12 using 2 M sodium hydroxide and 1 M hydrochloric acid. The remaining solution was aliquoted and snap frozen and stored at -80°C. The concentration of 1-pHis was determined to be 2.2 mg/mL by ^1^H NMR using 1,4-dioxane (0.001 g, 1 μL, 0.012 mmol) as the standard. 1-pHis (0.0084 g, 2% yield): *R*_F_ = 0.44 (65:8:22 EtOH/32% (wt/wt) NH_3_/H_2_O); ^1^H NMR (400 MHz) 2.96 (dd, *J* = 8.0, 15.5 Hz, 1H), 3.19 (dd, *J* = 5.0, 15.5 Hz, 1H), 3.84–3.90 (m, 1H), 6.72 (s, 1H), 7.73 (s, 1H); ^13^C NMR (101 MHz) 30.6 (s), 55.3 (s), 126.2 (d, *J* = 8.0 Hz), 130.4 (d, *J* = 3.5 Hz), 140.4 (d, *J* = 5.0 Hz), 181.9 (s); ^31^P NMR (162 MHz) - 5.61; m/z (ESI+): 236.0431 (MH+, 100% C_6_H_11_N_3_O_5_P requires 236.0400). The characterization data are comparable to the literature [18].

#### Synthesis of 2-amino-3-(1-phosphonoimidazol-4-yl)propanoic acid (3-(τ)-pHis)

L-Histidine (0.251 g, 1.62 mmol, 1.0 equiv.), potassium phosphoramidate (0.57 g, 4.21 mmol, 2.6 equiv.) and water (4.5 mL) were stirred at 25° C for 16 hours. 3-pHis was chromatographically purified as described below. The combined product fractions were concentrated by rotary evaporation at 25°C to approximately 8 ml whilst maintaining a pH between 10–12 using 2 M sodium hydroxide and 1 M hydrochloric acid. The remaining solution was aliquoted, snap frozen and stored at -80°C. The concentration of 3-pHis was determined to be 4.9 mg/mL by ^1^H NMR against 1,4-dioxane as the standard. 3-pHis (0.103 g, 27% yield): *R*_F_ = 0.40 (65:8:22 EtOH/32% (wt/wt) NH_3_/H_2_O); ^1^H NMR (400 MHz) 2.53 (dd, *J* = 9.0, 14.5 Hz, 1H), 2.79 (dd, *J* = 4.5, 14.5 Hz, 1H), 3.32–3.38 (m, 1H), 6.86 (s, 1H), 7.56 (s, 1H); ^13^C NMR (101 MHz) 32.6 (s), 56.0 (s), 117.8 (d, *J* = 5.5 Hz), 137.0 (d, *J* = 8.5 Hz), 139.0 (d, *J* = 5.0 Hz), 181.2 (s); ^31^P NMR (162 MHz) - 4.78; m/z (ESI+): 236.0431 (MH+, 100% C_6_H_11_N_3_O_5_P requires 236.0400). The characterization data are similar to the literature [18].

Flash chromatography was carried out at 4°C and a positive pressure of 0.68 atmospheres. The sample was loaded on to a pre-equilibrated (85:4:1 ethanol/32% (wt/wt) ammonia/water) silica gel column (4 cm diameter column with 27 cm of silica) and eluted with a mixture of ethanol, 32% (wt/wt) ammonia and water gradient. (Initially 300 mL of an 85:4:1 mixture was added: once 150 mL was eluted, 150 mL of a 75:4:10 mixture was added. After a further 150 mL was eluted, an additional 150 mL of this solvent was added. Subsequently the same cycle was repeated with solvent mixture ratio of 70:4:15 (300 mL), and 60:4:25 (300 mL), until the compound was eluted). The first 750 mL was run off and 15 mL fractions were collected. The product fractions were collected such that 5 fractions between the product and the first (for 1-pHis) and last (1-pHis) co-eluted 1- and 3-pHis mixture were discarded as analysed by TLC.

#### Synthesis of phosphorylated myoglobin (Myo-pHis), and dialysis

Myoglobin from equine heart (50 mg, 2.8 μM, 1 equiv.) was dissolved in water (4.5 mL). Potassium phosphoramidate (0.61 g, 4.5 mmol, 1585 equiv.) was added and the mixture was stirred at 25°C for 15 hours. The mixture was added to a Vivaspin 20 (10 kDa, MWCO) and concentrated down to ~1 mL by centrifugation (6°C, 3220 RCF). The solution was made up to 5 mL with dialysis buffer (0.1 M sodium carbonate/bicarbonate, pH 10.8) and the solution was again concentrated down to ~1 mL as described above; this step was repeated 7 times. The sample was made up to 2 mL using dialysis buffer. The Myo-pHis protein concentration was determined by Bradford assay and myoglobin was used to generate the standard curve. 10% (v/v) D_2_O was added to the Myo-pHis solution and analysed by ^31^P NMR spectroscopy. The sample was stable for weeks at 4°C and could be snap frozen and stored at 80°C (freeze thaw cycles were avoided). For ^31^P NMR spectroscopy of denatured Myo-pHis, urea (210 mg) was added to the Myo-pHis (500 μL) solution followed by D_2_O (50 μL) before analysis.

#### Acid treatment of Myo-pHis

The pH of Myo-pHis was reduced to ~pH 4 using glacial acetic acid and it was then heated at 90°C for 45 min. A precipitate was observed. Sodium carbonate (10 mg) and sodium hydrogen carbonate (1 mg) was added. To help dissolve the precipitate, urea (210 mg) and ethylenediaminetetraacetic acid disodium salt dihydrate (9 mg) were added and left overnight. 10% (v/v) D_2_O was added to the sample before analysis by ^31^P NMR spectroscopy.

#### Culturing of human bronchial epithelial (16HBE14o- or HBE) cell line

16HBE14o- cell line was cultured in 57 cm^2^ dishes in 10 mL full serum medium (Medium 199, supplemented with 10% (v/v) fetal bovine serum, 1.3 mM of L-glutamine, 80 μg/mL streptomycin, 80 units/mL penicillin) and incubated at 37°C in a 5% CO_2_ atmosphere. The medium was changed every 2–3 days by discarding the old medium, washing the cells with PBS (3 mL) and then replacing with fresh medium (10 mL). Once the 16HBE14o- cells were between 70–90% confluency, they were split or harvested.

#### Lysis of 16HBE14o- cells for Western blots

Media from 57 cm^2^ cell culture dishes with adherent 16HBE14o- cells was discarded and the cells were washed with PBS (5 mL). The cell culture dish was cooled on ice and any residual PBS was discarded. Cells were scraped into 100 μl ice cold lysis buffer (150 mM sodium chloride, 0.5% (wt/v) sodium deoxycholate, 1% (v/v) Triton X-100, 0.1% (wt/v) sodium dodecyl sulfate, 10 mM sodium fluoride, 5 mM sodium orthovanadate, 10 mM sodium pyrophosphate, complete protease inhibitor, 50 mM tris(hydroxymethyl)aminomethane-hydrochloride, pH 8.8). After centrifugation (4°C, 8950 RCF, 1 min), the supernatant was taken and used immediately or snap frozen and used within 3 days.

#### Western blot

Protein samples were treated with 5× sample buffer (10% (wt/v) lithium dodecyl sulfate, 40% (wt/v) glycerol, 0.02% (wt/v) bromophenol blue, 50 mM ethylenediaminetetraacetic acid disodium salt dihydrate, 500 mM dithiothreitol, 300 mM tris(hydroxymethyl)aminomethane-hydrochloride, pH 9.0) to give a 1× final concentration and then made up to 30 μL with 1× sample buffer. The samples were left at room temperature for 15 minutes and then loaded in to the 12% SDS-PAGE gel wells with a pH 8.8 stacking gel. The samples were resolved using resolving buffer (0.1% (w/v) sodium dodecyl sulfate, 192 mM Glycine, 25 mM tris(hydroxymethyl)aminomethane, pH 8.3) with the tank immersed in ice, at 120 V for the first 10 minutes and then 180 V for 50–60 minutes. The resolved proteins were immediately electro blotted onto a methanol activated PVDF membrane using transfer buffer (192 mM glycine, 25 mM tris(hydroxymethyl)aminomethane, pH 8.3) with the tank immersed in ice at 100 V for 1 hour. The following steps were carried out at room temperature. Blocking buffer (75 mL; 0.2% (v/v) gelatin from cold water fish skin, 165 mM sodium chloride, 0.05% (v/v) Tween 20, 10 mM tris(hydroxymethyl)aminomethane-hydrochloride, pH 8.0) was added over the membrane (~8 × 8 cm) in a covered tray (10 cm × 14 cm) and left for 1 hour on an orbital shaker. The solution was discarded. The membrane and primary antibody diluted in 10 mL blocking buffer was added to a 50 mL centrifuge vial and left for 1 hour on a tube roller. The solution was discarded, and the membrane was put back in the tray on an orbital shaker and washed (6 × 5 min) with 50 mL wash buffer (165 mM sodium chloride, 0.05% (v/v) Tween 20, 10 mM tris(hydroxymethyl)aminomethane-hydrochloride, pH 8.0). The membrane and secondary antibody diluted in 10 mL blocking buffer were added to a centrifuge vial and left for 1 hour on a tube roller. The solution was discarded, and the membrane was put back in the tray on an orbital shaker and washed (6 × 5 min) with 50 mL wash buffer. The membrane was then incubated in chemiluminescence solution for 1 minute. After draining off the excess chemiluminescence solution, the membrane was imaged.

#### Trypsinization of Myo-pHis and desalting under typical conditions

The method followed Chen *et al*. [23] with minor changes. Myo-pHis (20 mg) was buffer exchanged (3 cycles of buffer exchange) into dialysis buffer (0.1 M ammonium bicarbonate, pH 8.0) using a Vivaspin 20 (3 kDa MWCO) and made up to 1 mL. 1/50 (wt/wt) trypsin was added and the mixture was shaken (600 rpm) at 37° C for 16 hours. To half of the mixture, sodium carbonate (10 mg), sodium hydrogen carbonate (1 mg), and D_2_O (50 μL) was added and analyzed by ^31^P NMR spectroscopy.

A C-18 resin Sep-Pak cartridge (130 mg) was conditioned sequentially with acetonitrile (2.6 mL), 50% (v/v) acetonitrile, 0.1% trifluoroacetic acid (2.6 mL) and 0.1% trifluoroacetic acid (2.6 mL). The remaining sample (~500 μL) was made up to 2 mL using water. Half of the sample (1 mL) was acidified to pH 3 using aqueous 10% (v/v) trifluoroacetic acid and passed through the cartridge. The flow through was collected and passed through the cartridge again; this step was repeated once more. The resin was washed with 0.1% (v/v) trifluoroacetic acid (2.6 mL), and then extracted with 50% (v/v) acetonitrile, 0.1% (v/v) trifluoroacetic (0.6 mL). The other half of the sample (1 mL) was desalted in the same way using a fresh cartridge. The solvent from the combined fractions was removed under reduced pressure. The remaining residue was solubilized in 0.1 M sodium carbonate/hydrogen carbonate, pH 10.8 (500 μL), and analyzed by ^31^P NMR spectroscopy after the addition of 50 μL D_2_O. Protein concentration was checked using a BCA assay.

#### Trypsinization of Myo-pHis and desalting under milder conditions

The method followed Hardman *et al*. [2] with minor changes. Myo-pHis (20 mg) was buffer exchanged (3 cycles of buffer exchange) into dialysis buffer (0.1 M ammonium bicarbonate) using a Vivaspin 20 (3 kDa MWCO) and made up to 1 mL. The sample was treated with dithiothreitol (7 mM) and shaken (600 rpm) at 30° C for 20 minutes. After allowing the mixture to cool to room temperature, iodoacetamide (14 mM) was added and the mixture was left in the dark at room temperature. Dithiothreitol (3 mM) was added followed by 2% (wt/wt) trypsin and the mixture was shaken (600 rpm) at 30° C for 16 hours. To half of the mixture (~ 500 μL), sodium carbonate (10 mg), sodium hydrogen carbonate (1 mg), and D_2_O (50 μL) was added and analysed by ^31^P NMR spectroscopy.

A C-18 resin Sep-Pak cartridge (130 mg) was conditioned sequentially with methanol (2.6 mL), 50% (v/v) acetonitrile (2.6 mL) and water (2.6 mL). The remaining sample (~500 μL) was made up to 2 mL using water and half of the sample (1 mL) was passed through the cartridge. The flow through was collected and passed through the cartridge again; this step was repeated once more. The resin was washed with water (2.6 mL), and then extracted with 50% (v/v) acetonitrile (0.6 mL). The other half of the sample (1 mL) was desalted in the same way using a fresh cartridge. Both desalted samples were combined and solvent was removed under reduced pressure. The remaining residue was solubilized in 0.1 M sodium carbonate/hydrogen carbonate, pH 10.8 (500 μL), and analysed by ^31^P NMR spectroscopy after the addition of D_2_O.

#### Preparation of proteins from the 16HBE14o- cell lysate for ^31^P NMR spectroscopy

The lysis and dialysis procedure was done as quickly as possible. The medium was removed from five cell culture dishes (57 cm^2^) containing adhered 16HBE14o- cells between 70–90% confluent. The medium was discarded, and the cells were washed with PBS (10 mL). The petri dishes were cooled on ice and care was taken to remove as much PBS as possible. Ice cold lysis buffer (150 μL: 0.1 M sodium carbonate/bicarbonate, 7 M urea, pH 10.8) was added to each petri dish. Each petri dish was carefully shaken to evenly spread the lysis buffer. The cells were scraped into the lysis buffer and the lysate from each dish was transferred to another vial on ice. The lysis procedure was repeated three more times for a total of twenty cell culture dishes. For a cell count, five petri dishes were taken, and the cells were washed as described above. The combined cell lysate was sonicated on ice (45% amplitude 10 × 5 sec bursts, 59 sec rest between each burst). The lysate (~ 6 mL) was added to a Vivaspin 20 protein concentrator spin column and concentrated down to ~1.5 mL by centrifugation (6°C, 3220 RCF, ~2.5 hr). 1 mL dialysis buffer (7 M urea, 0.1 M sodium carbonate/bicarbonate, pH 10.8) was added, and the mixture was concentrated down as above to ~ 1.5 mL. This step was repeated 14 times. The sample finally was concentrated down to ~0.5 mL. 10% (v/v) D_2_O was added to a portion of this sample which was analysed by ^31^P NMR spectroscopy (for quantitative analysis 300 μL was used to accommodate the capillary containing the external standard). The 16HBE14o- cell lysate protein concentration was quantified by BCA assay, using BSA to generate the standard curve. The sample was stable for weeks at 4°C and could be snap frozen and stored at -80°C (freeze thaw cycles were avoided).

Cell counting: Each dish was treated with 2 mL of trypsin, at 37°C for 7 minutes. A cell scraper was used to help dislodge any weakly adhered cells. The cells in each petri dish were diluted with full serum medium (Medium 199 (including phenol red), supplemented with 10% (v/v) fetal bovine serum, 1.3 mM of L-glutamine, 80 μg/mL streptomycin, 80 units/mL penicillin) to give a total volume of 10 mL and transferred to a separate vial. In a triplicate cell count, 10 μL was taken from each cell suspension to determine the average cell count using a hemocytometer. The average cell count of each cell suspension was then averaged between the five separate samples, to give an average cell count.

#### Trypsinization of proteins from 16HBE14o- cell lysate and desalting under typical conditions

The method followed Chen *et al*. [23] with minor changes. The prepared 16HBE14o- cell lysate (sample volume used in the previous ^31^P NMR spectroscopy step) was buffer exchanged (3 cycles of buffer exchange) into dialysis buffer (0.1 M ammonium bicarbonate, pH 8.0) using a Vivaspin 20 (3 kDa MWCO) and made up to 1 mL. 1/50 (wt/wt) trypsin was added and the mixture was shaken (600 rpm) at 37° C for 16 hours. The sample was divided in two and to one half of the sample sodium carbonate (5 mg), sodium bicarbonate (0.5 mg) and 10% (v/v) D_2_O was added and analysed by ^31^P NMR spectroscopy. The other half of the sample was desalted as described in the “Trypsinization of Myo-pHis and desalting under typical conditions” section.

#### Trypsinization of proteins from the 16HBE14o- cell lysate and desalting under milder conditions

The method followed Hardman *et al*. [2] with minor changes. The medium was removed from five cell culture dishes (57 cm^2^) containing adhered 16HBE14o- cells between 70–90% confluent. The medium was discarded, and the cells were washed with PBS (10 mL). The petri dishes were cooled on ice and care was taken to remove as much PBS as possible. Ice cold lysis buffer (150 μL: 50 mM ammonium bicarbonate, 8 M urea, complete protease inhibitor cocktail, pH 8.0) was added to each petri dish. Each petri dish was carefully shaken to evenly spread the lysis buffer. The cells were scraped into the lysis buffer and the lysate from each dish was transferred to another vial on ice. The lysis procedure was repeated once more for a total of 10 cell culture dishes. The combined cell lysate was sonicated on ice (35% amplitude, 10 × 5 sec bursts, 59 sec between each burst). The lysate was treated with dithiothreitol (7 mM) and shaken (600 rpm) at 30° C for 20 minutes. After allowing the mixture to cool to room temperature, iodoacetamide (14 mM) was added and the mixture was left in the dark at room temperature. Dithiothreitol (3 mM) was added and the mixture was diluted to 2 M urea using 50 mM ammonium bicarbonate, pH 8.0. 2% (wt/wt) trypsin was added and the mixture was shaken (600 rpm) at 30° C for 16 hours. A C-18 resin Sep-Pak cartridge (130 mg) was conditioned sequentially with methanol (2.6 mL), 50% (v/v) acetonitrile (2.6 mL) and water (2.6 mL). The remaining sample (~500 μL) was made up to 2 mL using water and half of the sample (1 mL) was passed through the cartridge. The flow through was collected and passed through the cartridge again; this step was repeated once more. The resin was washed with water (2.6 mL), and then extracted with 50% (v/v) acetonitrile (0.6 mL). The other half of the sample (1 mL) was desalted in the same way using a fresh cartridge. Both desalted samples were combined and solvent was removed under reduced pressure. The remaining residue was solubilized in 0.1 M sodium carbonate/hydrogen carbonate, pH 10.8 (500 μL) and analysed by ^31^P NMR spectroscopy after the addition of 10% (v/v) D_2_O.

#### DNA extraction and precipitation of proteins from the 16HBE14o- cell lysate

The method followed Antonioli *et al*. with slight modifications [35]. 16HBE14o- cells (700 μL, 18.1 mg of protein) were sonicated on ice (45% amplitude 10 × 5 sec bursts, 59 sec between each burst) in lysis buffer (30 mM tris(hydroxymethyl)aminomethane hydrochloride, 50 mM sodium chloride, complete protease inhibitor, pH 9.0). The pH was raised to pH 10 using 2 M NaOH. The lysate was centrifuged to pellet any debris. The supernatant was removed and 1.4 mL DNA extraction solution (phenol/chloroform/isoamyl alcohol 25:24:1) was added. The mixture was vortexed and left on ice for 5 minutes and then centrifuged (4°C, 17000 RCF, 10 min). The upper aqueous and the bottom organic phase were carefully removed. Acetone (3 ml) was added to the remaining precipitate. The precipitate was centrifuged, and the supernatant was removed. The precipitate was allowed to air dry and then solubilized in in 1.5 mL dialysis buffer (8 M urea, 0.1 M sodium carbonate/bicarbonate, pH 10.8). The mixture was added to a Vivaspin 20 protein spin concentrator and concentrated down to ~ 1.0 mL by centrifugation (6°C, 3220 RCF, ~2.5 hr). 1 mL dialysis buffer was added, and the mixture was concentrated down as above to ~ 1.0 mL. This step was repeated 7 times. The sample was concentrated down to ~ 0.5 mL and 10% (v/v) D_2_O was added before analysis by ^31^P NMR spectroscopy.

#### Precipitation of proteins from the 16HBE14o- cell lysate

The method followed Potel *et al*. [29] with minor modification. Lysed cells were prepared. Total lysate volume after sonication: 6.5 mL. Methanol (13 mL), chloroform (3.3 mL), and water (10 mL) were sequentially added to the lysate and followed by vigorous vortexing after the addition of each solution. The mixture was centrifuged (6°C, 3220 RCF, 10 min) and the upper layer was discarded. Methanol (10 mL) was added and the mixture was vortexed and then centrifuged (6°C, 3220 RCF, 10 min). The supernatant was discarded. The precipitate was dissolved in 0.1 M sodium carbonate/bicarbonate, pH 10.8 and 10% (v/v) D_2_O was added before ^31^P NMR spectroscopy.

#### Chemical phosphorylation

The proteins from the 16HBE14o- cells prepared from the “DNA extraction” procedure (500 μL) were treated with potassium phosphoramidate (68 mg, 0.5 mmol). The mixture was turned end over end at room temperature for 15 minutes. Dialysis buffer (400 μL) and water (200 μL) was added and the mixing was continued at room temperature for a further 1 hour. The mixture was made up to 3.5 mL with dialysis buffer (8 M urea, 0.1 M sodium carbonate/bicarbonate, pH 10.8). The mixture was added to a Vivaspin 20 protein concentrator spin column and concentrated down to ~ 1.5 mL by centrifugation (6°C, 3220 RCF, ~2.5 hr). 1 mL dialysis buffer was added, and the mixture was concentrated down as above to ~ 1.5 mL. This step was repeated 7 times. The sample was finally concentrated down to ~ 0.5 mL and 10% (v/v) D_2_O was added before analysis by ^31^P NMR spectroscopy.

#### Acid treatment of proteins from the 16HBE14o- cell lysate

The pH of proteins from the 16HBE14o- cell lysate in 91 mM sodium carbonate/bicarbonate, 6.4 M urea, 10% (v/v) D_2_O was reduced to ~pH 4 using glacial acetic acid and it was heated at 90°C for 45 min. A precipitate was observed. Sodium carbonate (10 mg) and sodium hydrogen carbonate (1 mg) was added. 10% (v/v) D_2_O was added to the sample before analysis by ^31^P NMR spectroscopy.

## Supporting information

S1 Fig ^31^P NMR spectra of phosphoamino acids in 6.4 M urea, 91 mM Na_2_CO_3_/NaHCO_3_, 10% (v/v) D_2_O, pH 10.8. **a**) 1-(π)-pHis; **b**) 3-(τ)-pHis. The minor signal at—5.63 ppm is a suspected rotamer of π-pHis: π-pHis in D_2_O, pH 10–12 gives only one signal at– 5.61 ppm); **c**) pSer; **d**) pThr; **e**) pTyr; and **f**) inorganic phosphate (Pi).
(TIF)

S2 Fig ^31^P NMR spectra of (top) Myo-pHis, and (bottom) Myo-pHis after acid treatment. (top) Myo-pHis (18.2 mg/mL) in 91 mM Na_2_CO_3_/NaHCO_3_, 10% (v/v) D_2_O, pH 10.8; (bottom) same Myo-pHis sample after acidification with glacial acetic acid to ~ pH 4 and heating at 90°C for 45 min. Na_2_CO_3_, NaHCO_3_, urea, Na_2_EDTA.2H_2_O and 10% (v/v) D_2_O were added before ^31^P NMR spectroscopy analysis.
(TIF)

S3 Fig Validation of pHis antibody (ab2317090) by Western blot of acid or base treated 16HBE14o- cell lysate. Western blot of 16HBE14o- cell lysate (100 μg of protein) using pHis antibody (ab2317090) before and after treatment with acid (acetic acid pH 7.0 at 90°C for 45 min) or base (NaOH 0.1 M final concentration at room temp. for 15 min). These results are similar to reports that have used similar validations on pHis antibodies [28,33].
(TIF)

S4 Fig ^31^P NMR spectra of pSer and pThr after two days at pH 10.8. **a**) pSer after 2 days at room temp. in 0.22 M Na_2_CO_3_/NaHCO_3_, 10% (v/v) D_2_O, pH 10.8; **b**) pThr after 2 days at room temp. in 0.22 M Na_2_CO_3_/NaHCO_3_, 10% (v/v) D_2_O, pH 10.8.
(TIF)

S5 Fig ^31^P NMR spectra of 16HBE14o- cell lysate before and after buffer exchange. All 16HBE14o- cells lysate samples are in 10% (v/v) D_2_O. **i)** 16HBE14o- cell lysate after lysis in 0.1 M Na_2_CO_3_/NaHCO_3_, 7 M urea, pH 10.8, and sonication; **ii**) same sample after concentration using a Vivaspin 20 (3 kDa, MWCO) but without exchanging buffer; **iii**) same sample after concentration and seven cycles of buffer exchange into 0.1 M Na_2_CO_3_/NaHCO_3_, 7 M urea, pH 10.8 using a Vivaspin 20 (3 kDa, MWCO); **iv**) same sample after concentration and fourteen cycles of buffer exchange into 0.1 M Na_2_CO_3_/NaHCO_3_, 7 M urea, pH 10.8 using a Vivaspin 20 (3 kDa, MWCO). The absence of the signal from inorganic phosphate suggests that buffer exchange was complete. Further buffer exchange cycles gave no further change in the spectrum.
(TIF)

S6 Fig ^31^P NMR spectrum of proteins from 16HBE14o- cell lysate prepared by protein precipitation. DNA was extracted following [35]. No signal from pHis remains.
(TIF)

S7 Fig ^31^P NMR spectrum of chemically phosphorylated proteins from the 16HBE14o- cell lysate. Proteins from 16HBE14o- cell lysate (S6 Fig) were chemically phosphorylated with 1 M potassium phosphoramidate at room temperature for 1 h, in 0.1 Na_2_CO_3_/NaHCO_3_, 8 M urea 10% (v/v) D_2_O, pH 10.8. The sample was subsequently buffer exchanged into 0.1 Na_2_CO_3_/NaHCO_3_, 8 M urea, pH 10.8. Potassium phosphoramidate is known to selectively phosphorylate His under neutral conditions [21] and phosphorylate Lys under more basic conditions [21]. The above ^31^P NMR spectrum suggests under the conditions used potassium phosphoramidate can phosphorylate other nucleophiles. Chemical shift assignments were made using literature values [30–32]. Orthophosphate diesters include DNA, RNA, and phospholipids. New signals previously not present include pHis, Pi, pLys, phosphonates and polyphosphates.
(TIF)

S8 Fig ^31^P NMR spectrum of proteins from the 16HBE14o- cell lysate before and after acid treatment. **i**) proteins from the 16HBE14o- cell lysate in 91 mM Na_2_CO_3_/NaHCO_3_, 6.4 M urea, 10% (v/v) D_2_O, pH 10.8 prepared as Fig 3; **ii**) same sample after acid treatment with acetic acid to ~pH 4 and heating at 90°C for 45 min. Na_2_CO_3_, NaHCO_3_, and 10% (v/v) D_2_O were added before ^31^P NMR spectroscopy analysis.
(TIF)

S9 Fig Triplicate ^31^P NMR analysis of proteins from 16HBE14o- cell lysate. ^31^P NMR spectrum of proteins from 16HBE14o- cell lysate **a**) 15.6 mg/mL; **b**) 15.1 mg/mL; **c**) 15.4 mg/mL in 91 mM Na_2_CO_3_/NaHCO_3_, 6.4 M urea, 10% (v/v) D_2_O, pH 10.8. 16HBE14o- cells were lysed on ice in 0.1 M Na_2_CO_3_/NaHCO_3_, 7 M urea, pH 10.8. The lysate was immediately sonicated and buffer exchanged into 0.1 M Na_2_CO_3_/NaHCO_3_, 7 M urea, pH 10.8. pSer and pThr signal assignments were made using literature chemical shift values [30,31].
(TIF)

S10 Fig Triplicate quantitative ^31^P NMR spectroscopy analysis of proteins from 16HBE14o- cell lysate. ^31^P NMR spectrum of proteins from the 16HBE14o- cell lysate using an external standard capillary to give a reference for corresponding ^31^P NMR spectra. Signal assignments were made using literature chemical shift values [30–32]. Note that chemical shift values have changed from those in S9 Fig because of the two different lock solvents used in the sample and external standard: D_2_O was used in the sample and CDCl_3_ was used in the external standard capillary.
(TIF)

S11 Fig ^31^P NMR spectrum of proteins from 16HBE14o- cell lysate after trypsinization and desalting following Hardman *et al*. **[2].** 16HBE14o- cells were lysed in 50 mM NH_4_HCO_3_, 8 M urea, complete protease inhibitor cocktail, pH 8.0. The lysate was sonicated before reduction with DTT (3 mmol), alkylation with IAA (14 mmol) and subsequent dilution to 2 M urea using 50 mM NH_4_HCO_3_, pH 8.0 and treated with 2% (wt/wt) trypsin, at 30°C for 16 h. The sample was subsequently desalted. Na_2_CO_3_, NaHCO_3_ and D_2_O were added before ^31^P NMR spectroscopy analysis.
(TIF)

S1 Table Quantitation of pHis and pSer/pThr from 16HBE14o- cell lysate. (TIF)

S2 Table Average amount of pHis, pSer/pThr, and polyphosphate per 16HBE14o- cell. (TIF)

S1 File Calculation of average number of pHis residues per myoglobin protein. (TIF)
