# Supplementary figures and images for: Quantitation of phosphohistidine in proteins in a mammalian cell line by 31P NMR
Source: PLoS One. 2022 Sep 1;17(9):e0273797. doi: 10.1371/journal.pone.0273797 (PMC9436146; doi:10.1371/journal.pone.0273797)

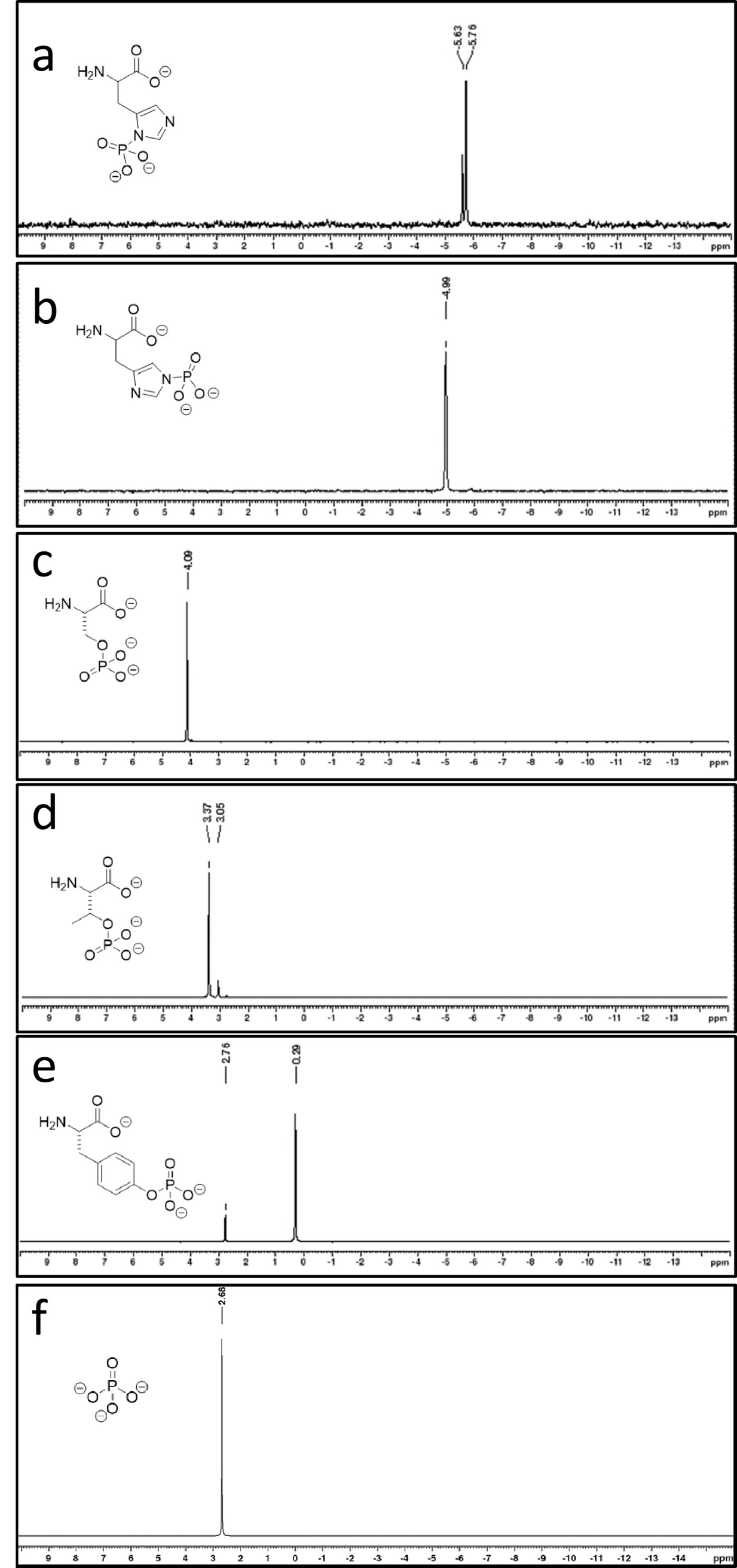

Supplement: S1 Fig — a) 1-(π)-pHis; b) 3-(τ)-pHis. The minor signal at—5.63 ppm is a suspected rotamer of π-pHis: π-pHis in D2O, pH 10–12 gives only one signal at– 5.61 ppm); c) pSer; d) pThr; e) pTyr; and f) inorganic phosphate (Pi). (TIF) [file pone.0273797.s001.tif]

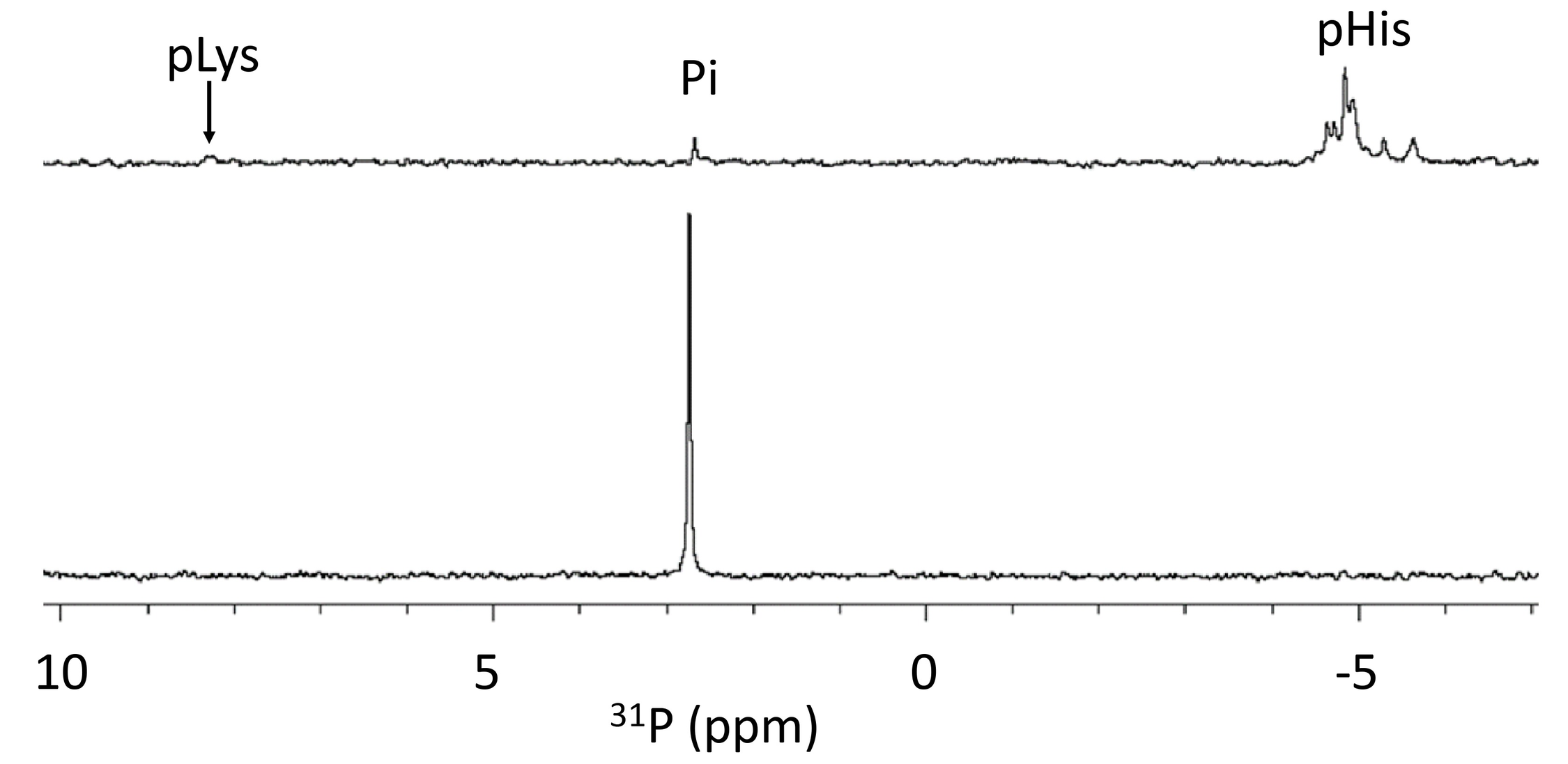

Supplement: S2 Fig — (top) Myo-pHis (18.2 mg/mL) in 91 mM Na2CO3/NaHCO3, 10% (v/v) D2O, pH 10.8; (bottom) same Myo-pHis sample after acidification with glacial acetic acid to ~ pH 4 and heating at 90°C for 45 min. Na2CO3, NaHCO3, urea, Na2EDTA.2H2O and 10% (v/v) D2O were added before 31P NMR spectroscopy analysis. (TIF) [file pone.0273797.s002.tif]

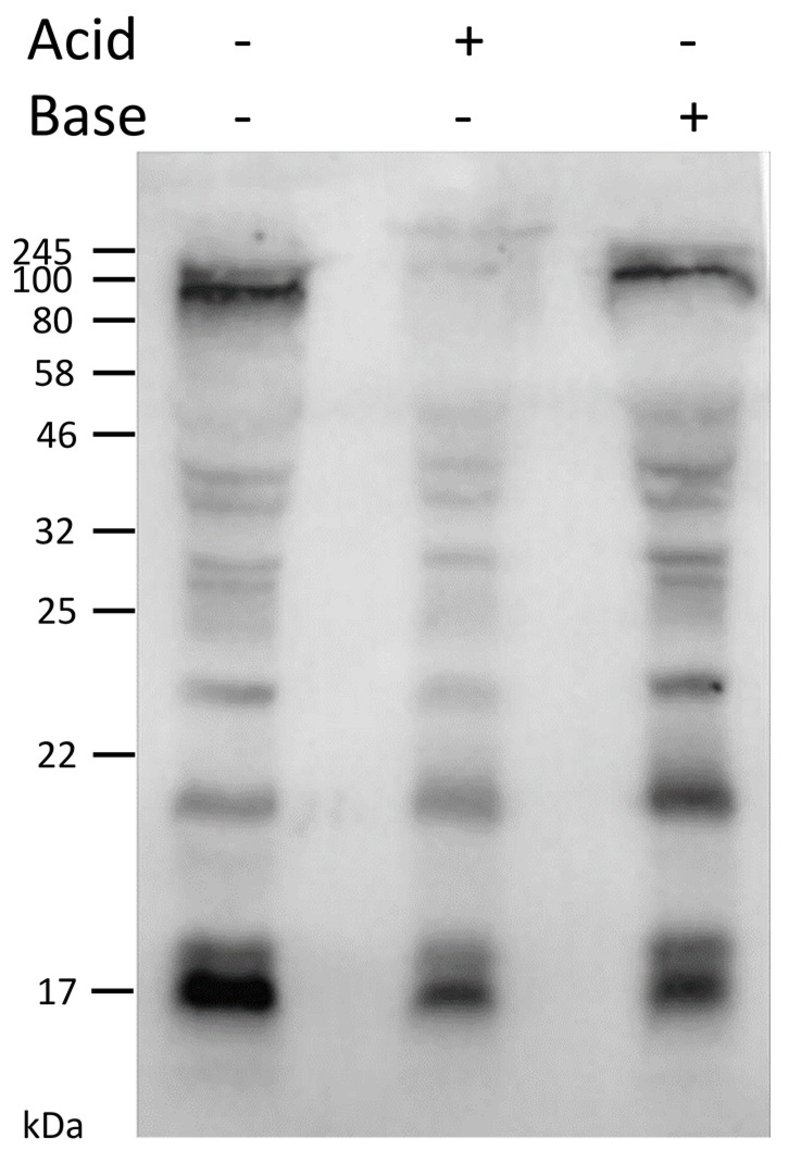

Supplement: S3 Fig — Western blot of 16HBE14o- cell lysate (100 μg of protein) using pHis antibody (ab2317090) before and after treatment with acid (acetic acid pH 7.0 at 90°C for 45 min) or base (NaOH 0.1 M final concentration at room temp. for 15 min). These results are similar to reports that have used similar validations on pHis antibodies [28,33]. (TIF) [file pone.0273797.s003.tif]

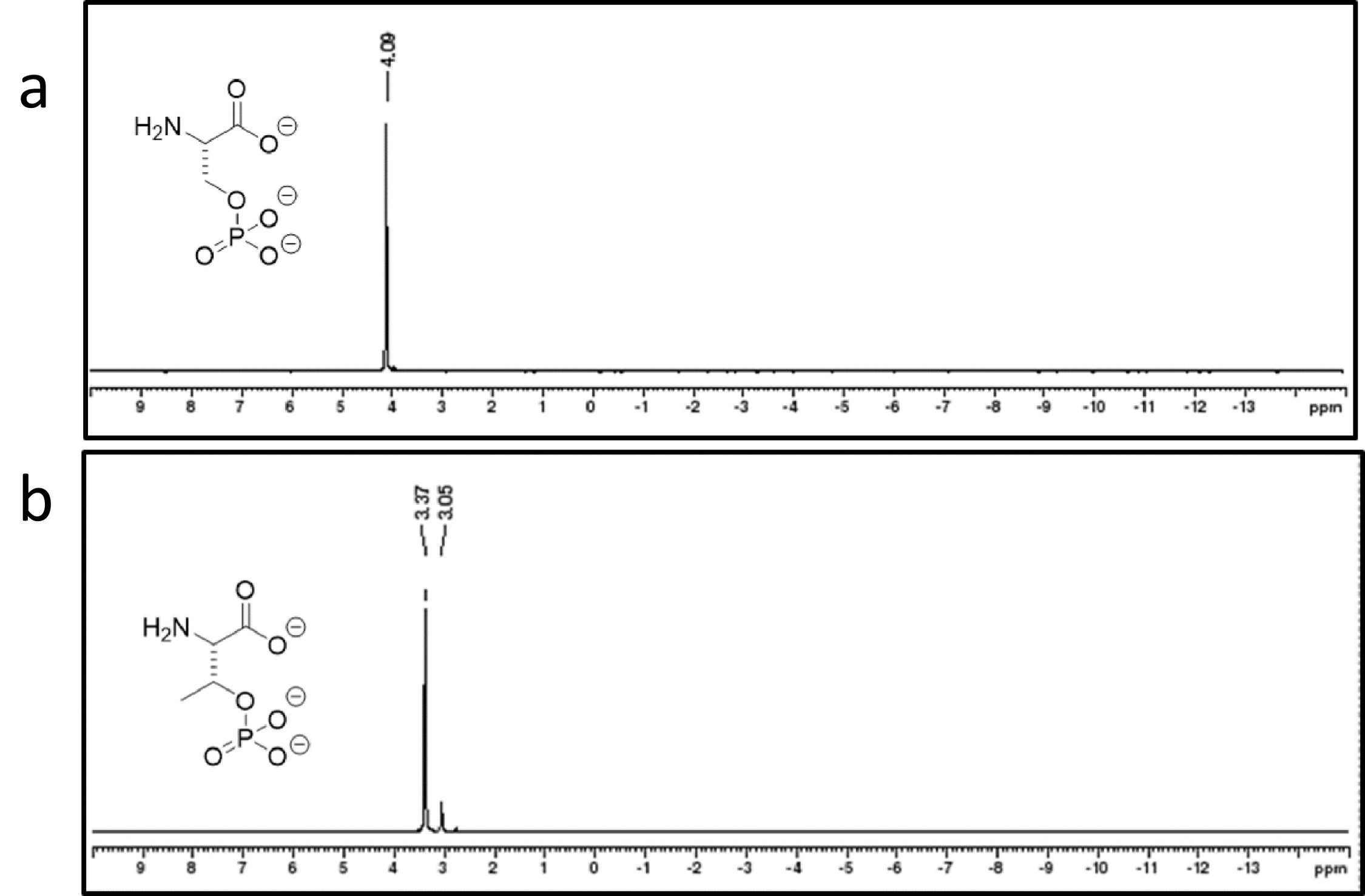

Supplement: S4 Fig — a) pSer after 2 days at room temp. in 0.22 M Na2CO3/NaHCO3, 10% (v/v) D2O, pH 10.8; b) pThr after 2 days at room temp. in 0.22 M Na2CO3/NaHCO3, 10% (v/v) D2O, pH 10.8. (TIF) [file pone.0273797.s004.tif]

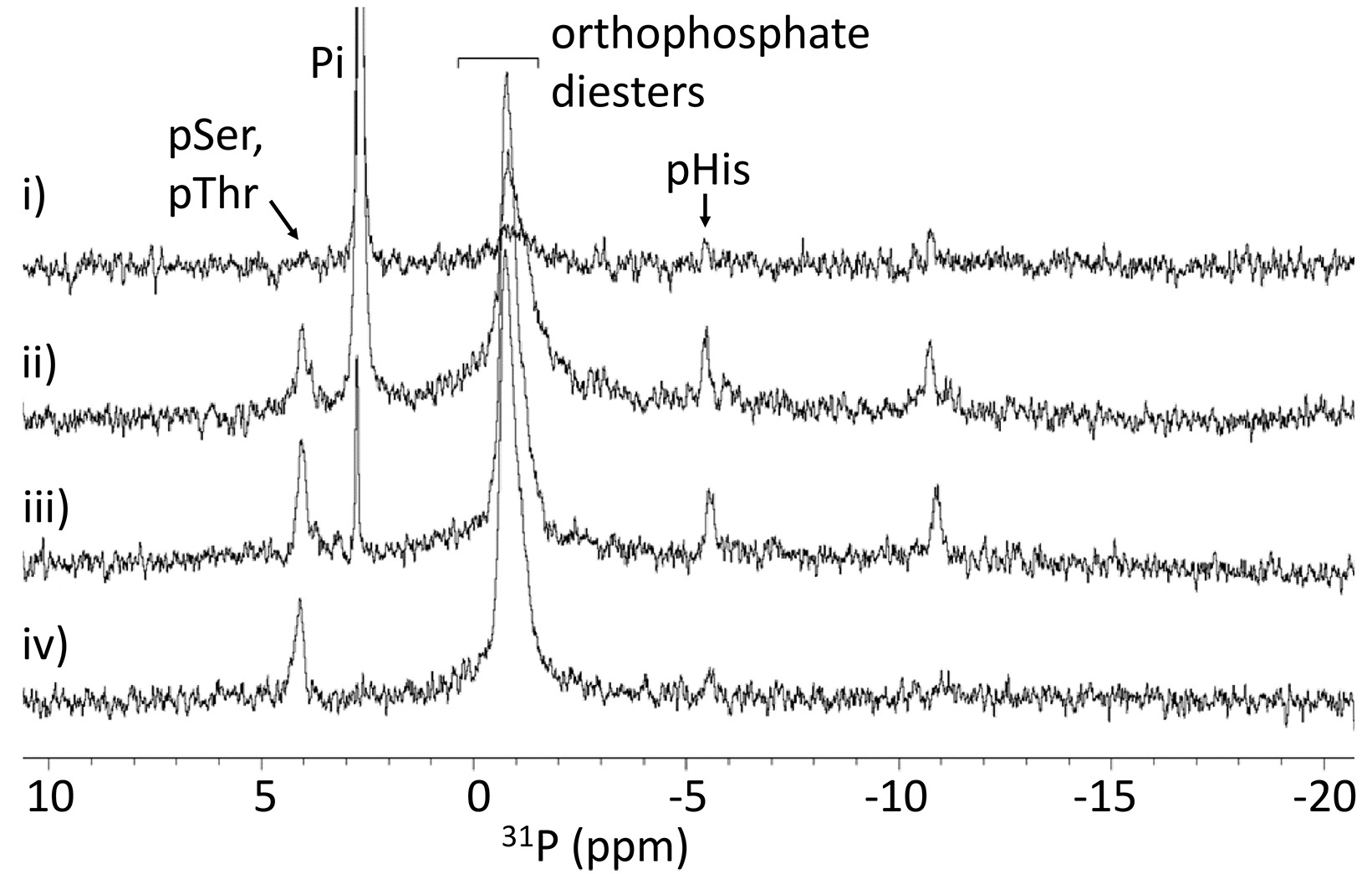

Supplement: S5 Fig — All 16HBE14o- cells lysate samples are in 10% (v/v) D2O. i) 16HBE14o- cell lysate after lysis in 0.1 M Na2CO3/NaHCO3, 7 M urea, pH 10.8, and sonication; ii) same sample after concentration using a Vivaspin 20 (3 kDa, MWCO) but without exchanging buffer; iii) same sample after concentration and seven cycles of buffer exchange into 0.1 M Na2CO3/NaHCO3, 7 M urea, pH 10.8 using a Vivaspin 20 (3 kDa, MWCO); iv) same sample after concentration and fourteen cycles of buffer exchange into 0.1 M Na2CO3/NaHCO3, 7 M urea, pH 10.8 using a Vivaspin 20 (3 kDa, MWCO). The absence of the signal from inorganic phosphate suggests that buffer exchange was complete. Further buffer exchange cycles gave no further change in the spectrum. (TIF) [file pone.0273797.s005.tif]

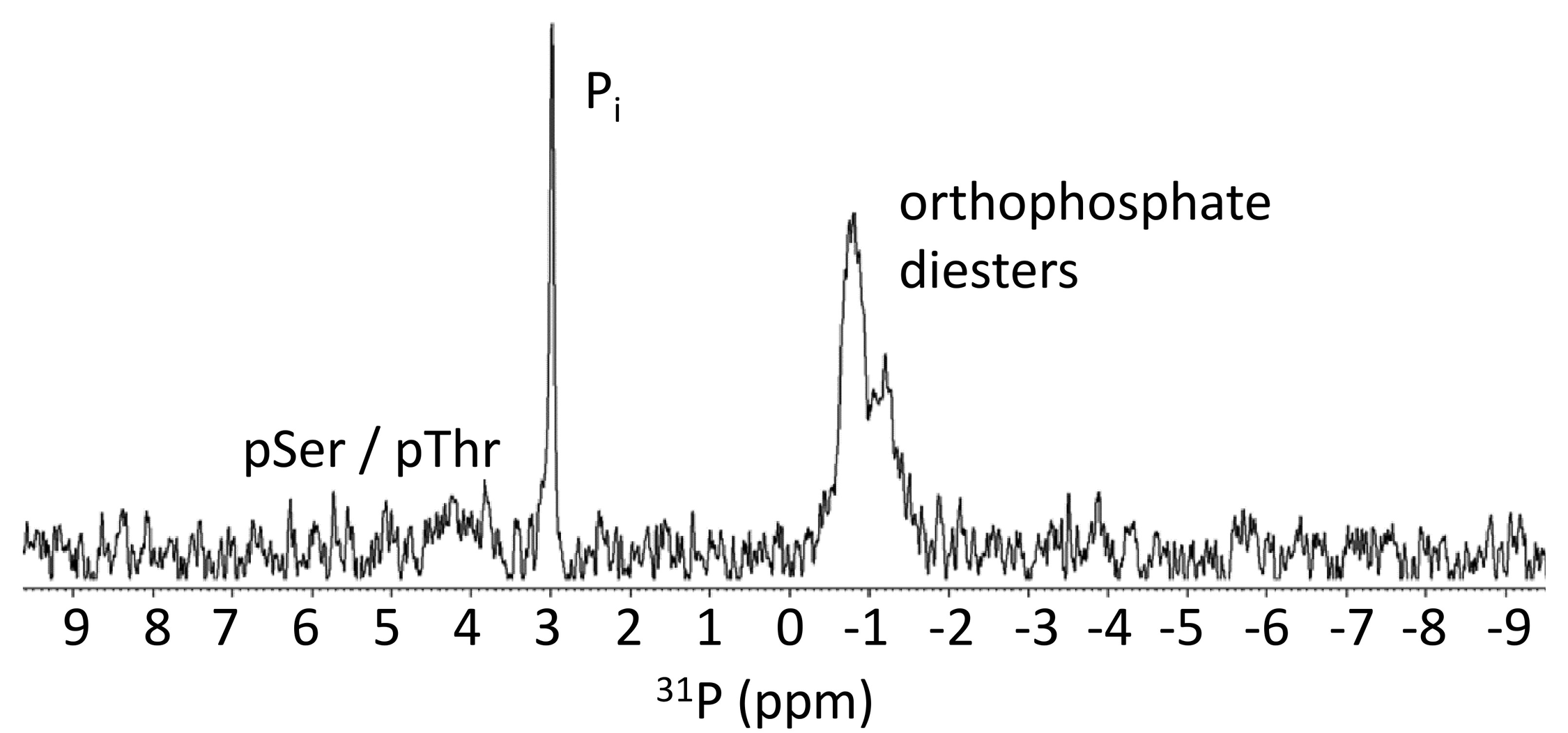

Supplement: S6 Fig — DNA was extracted following [35]. No signal from pHis remains. (TIF) [file pone.0273797.s006.tif]

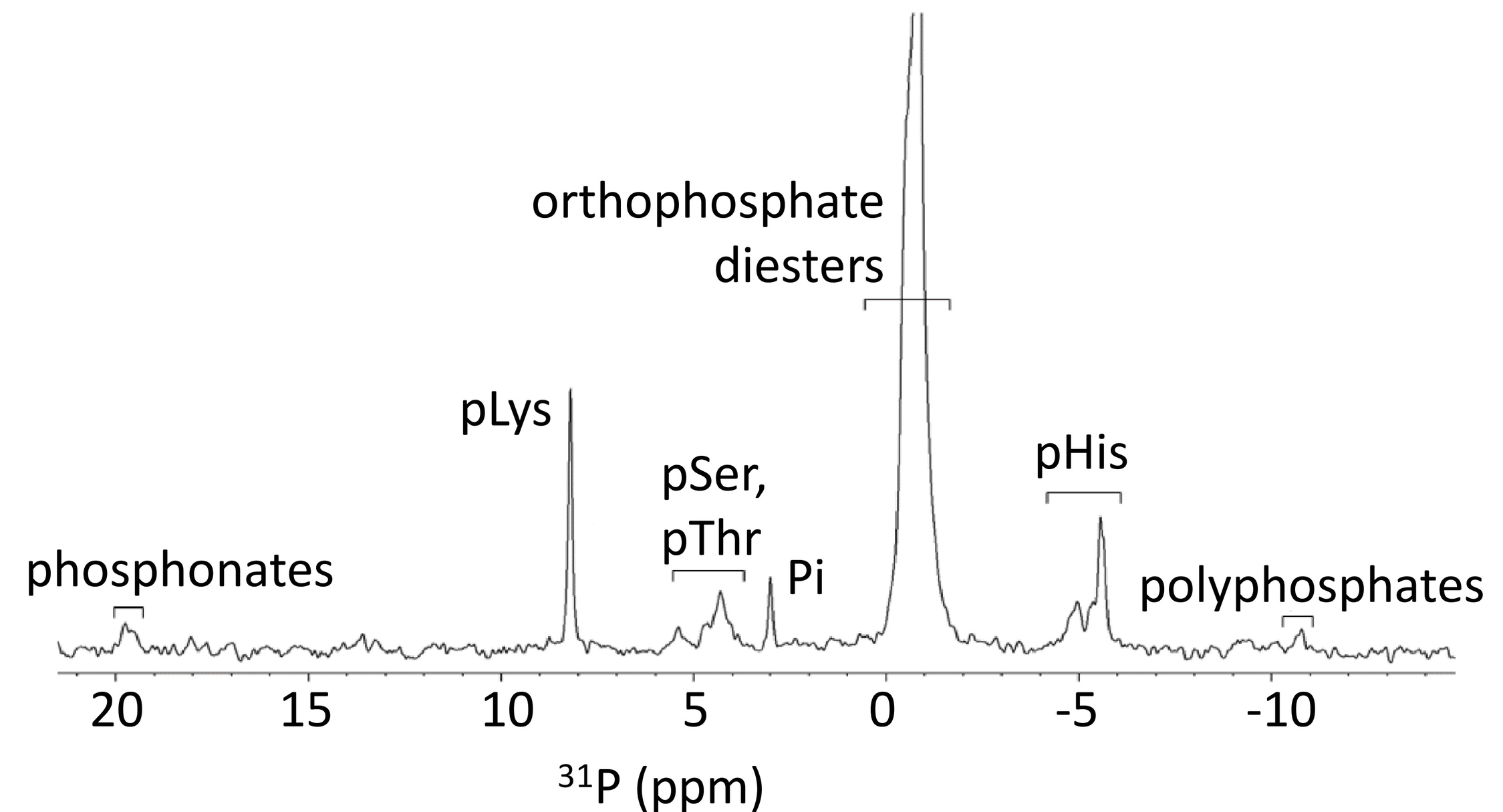

Supplement: S7 Fig — Proteins from 16HBE14o- cell lysate (S6 Fig) were chemically phosphorylated with 1 M potassium phosphoramidate at room temperature for 1 h, in 0.1 Na2CO3/NaHCO3, 8 M urea 10% (v/v) D2O, pH 10.8. The sample was subsequently buffer exchanged into 0.1 Na2CO3/NaHCO3, 8 M urea, pH 10.8. Potassium phosphoramidate is known to selectively phosphorylate His under neutral conditions [21] and phosphorylate Lys under more basic conditions [21]. The above 31P NMR spectrum suggests under the conditions used potassium phosphoramidate can phosphorylate other nucleophiles. Chemical shift assignments were made using literature values [30–32]. Orthophosphate diesters include DNA, RNA, and phospholipids. New signals previously not present include pHis, Pi, pLys, phosphonates and polyphosphates. (TIF) [file pone.0273797.s007.tif]

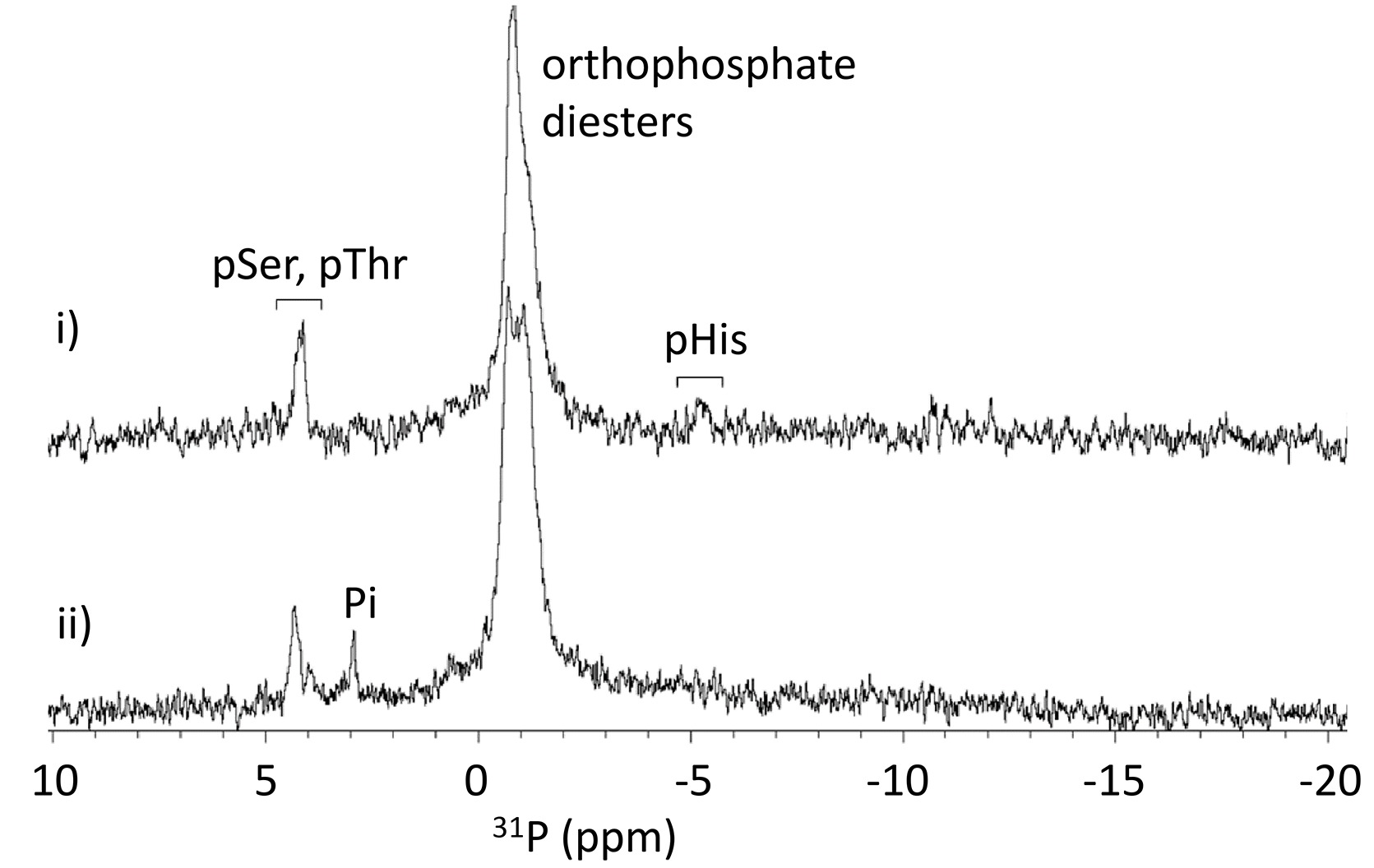

Supplement: S8 Fig — i) proteins from the 16HBE14o- cell lysate in 91 mM Na2CO3/NaHCO3, 6.4 M urea, 10% (v/v) D2O, pH 10.8 prepared as Fig 3; ii) same sample after acid treatment with acetic acid to ~pH 4 and heating at 90°C for 45 min. Na2CO3, NaHCO3, and 10% (v/v) D2O were added before 31P NMR spectroscopy analysis. (TIF) [file pone.0273797.s008.tif]

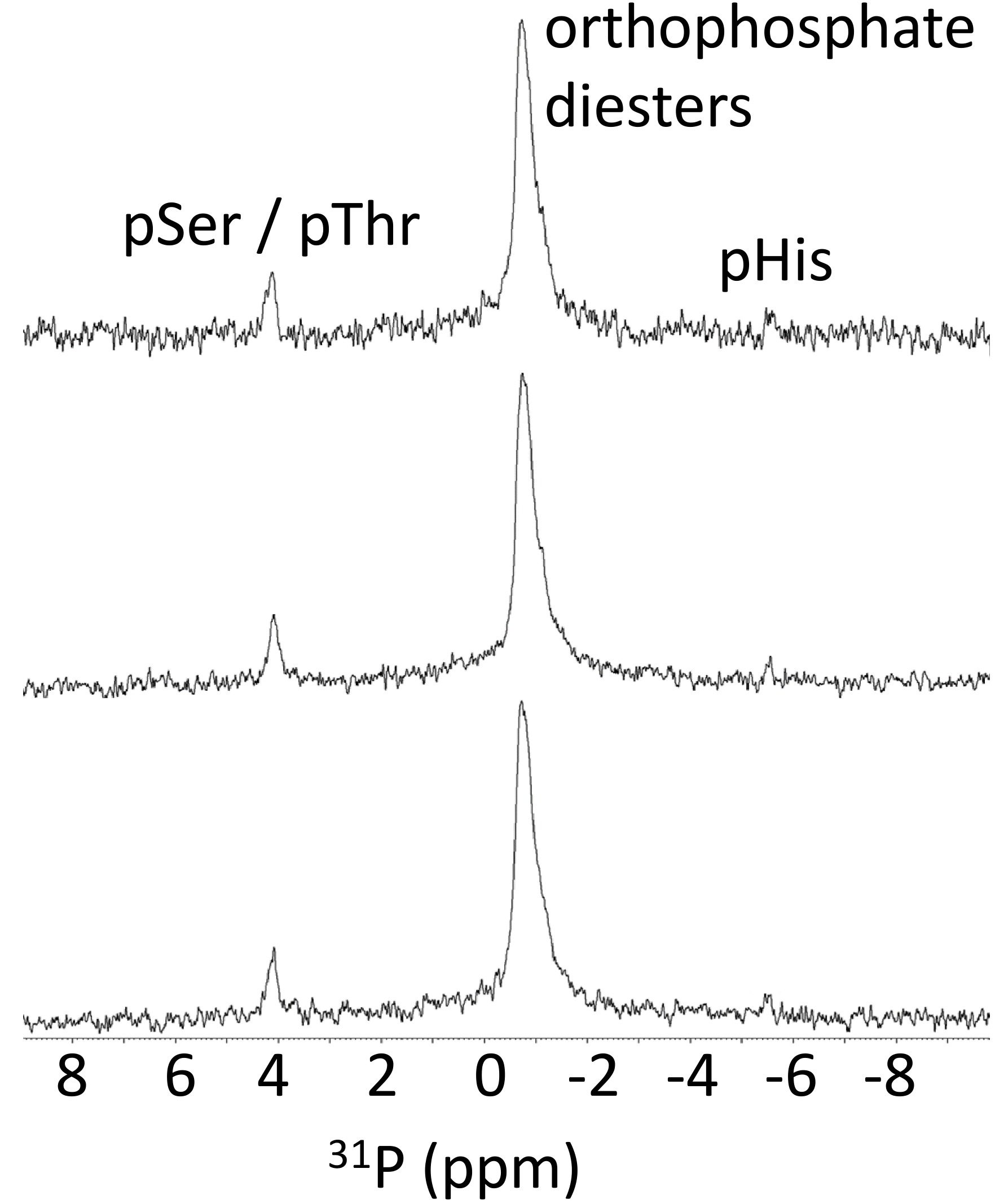

Supplement: S9 Fig — 31P NMR spectrum of proteins from 16HBE14o- cell lysate a) 15.6 mg/mL; b) 15.1 mg/mL; c) 15.4 mg/mL in 91 mM Na2CO3/NaHCO3, 6.4 M urea, 10% (v/v) D2O, pH 10.8. 16HBE14o- cells were lysed on ice in 0.1 M Na2CO3/NaHCO3, 7 M urea, pH 10.8. The lysate was immediately sonicated and buffer exchanged into 0.1 M Na2CO3/NaHCO3, 7 M urea, pH 10.8. pSer and pThr signal assignments were made using literature chemical shift values [30,31]. (TIF) [file pone.0273797.s009.tif]

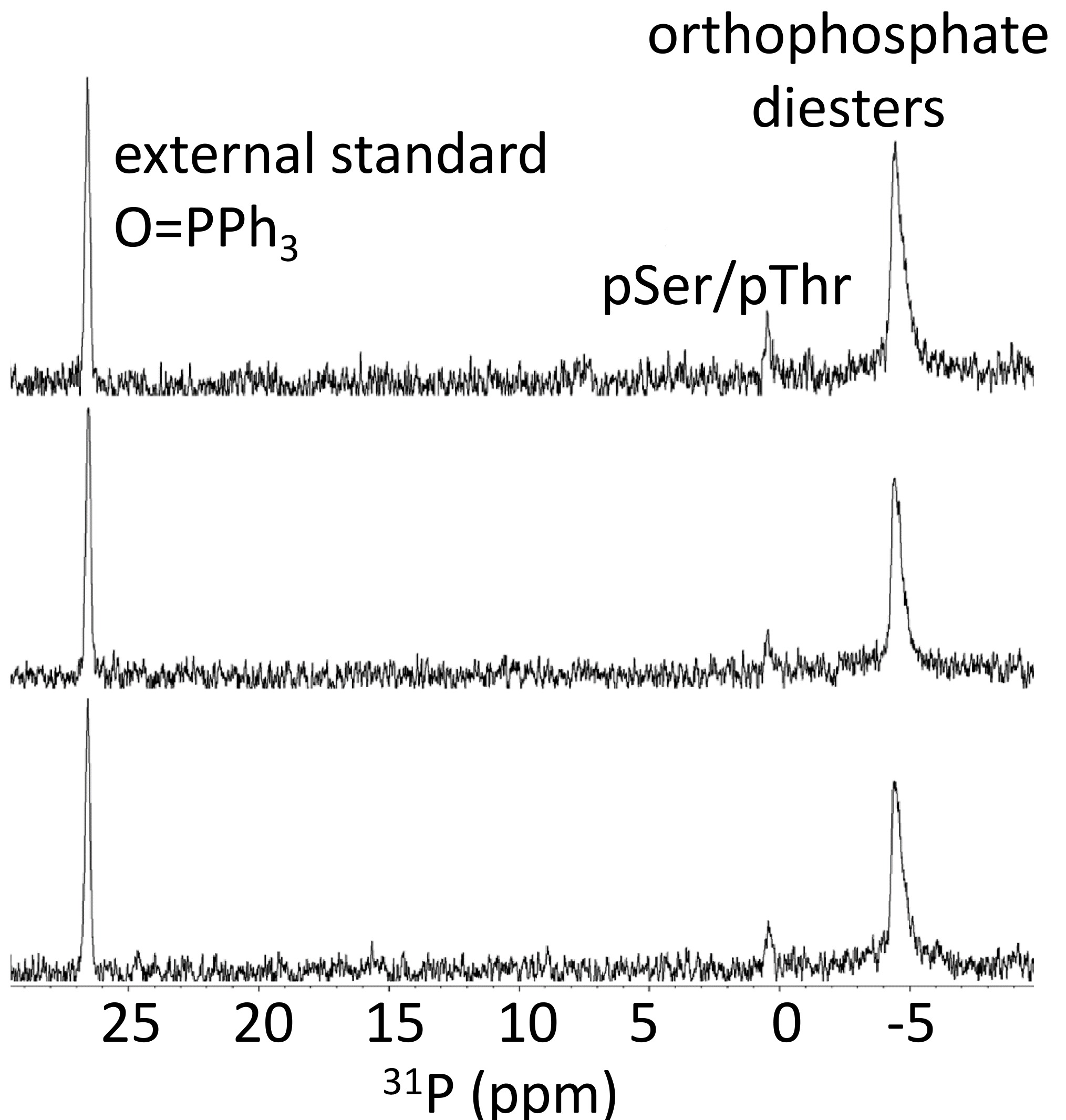

Supplement: S10 Fig — 31P NMR spectrum of proteins from the 16HBE14o- cell lysate using an external standard capillary to give a reference for corresponding 31P NMR spectra. Signal assignments were made using literature chemical shift values [30–32]. Note that chemical shift values have changed from those in S9 Fig because of the two different lock solvents used in the sample and external standard: D2O was used in the sample and CDCl3 was used in the external standard capillary. (TIF) [file pone.0273797.s010.tif]

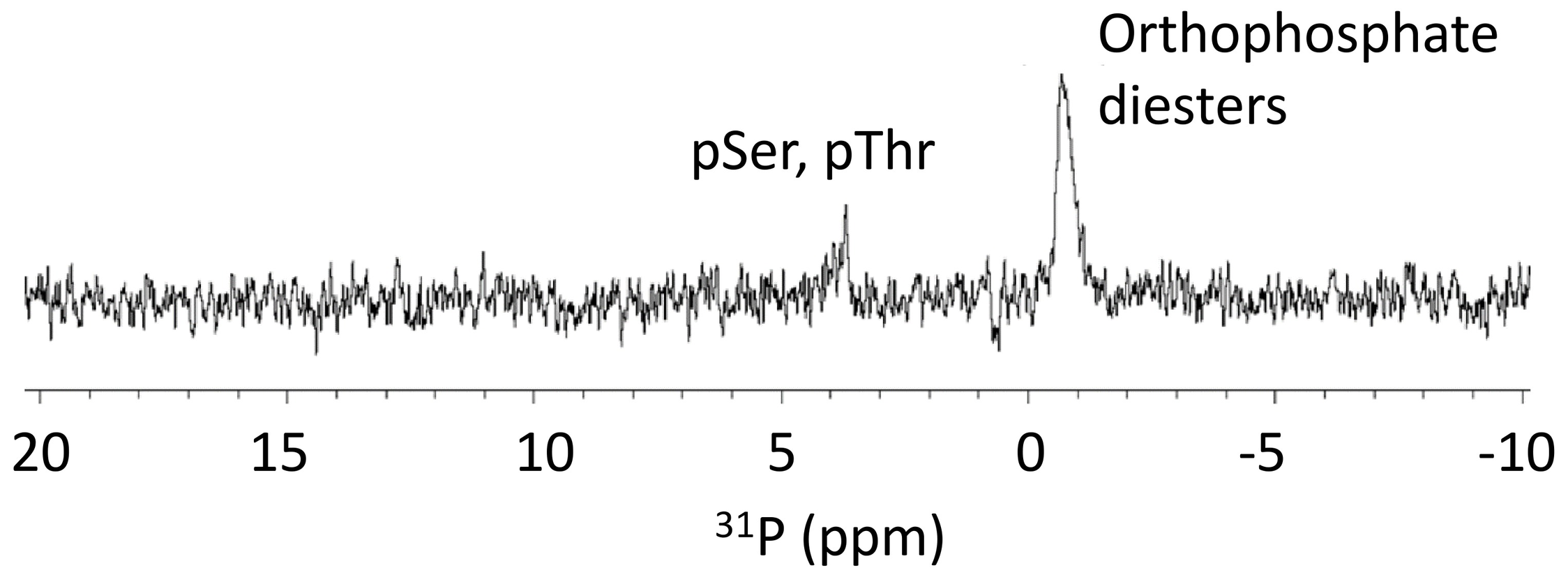

Supplement: S11 Fig — [2]. 16HBE14o- cells were lysed in 50 mM NH4HCO3, 8 M urea, complete protease inhibitor cocktail, pH 8.0. The lysate was sonicated before reduction with DTT (3 mmol), alkylation with IAA (14 mmol) and subsequent dilution to 2 M urea using 50 mM NH4HCO3, pH 8.0 and treated with 2% (wt/wt) trypsin, at 30°C for 16 h. The sample was subsequently desalted. Na2CO3, NaHCO3 and D2O were added before 31P NMR spectroscopy analysis. (TIF) [file pone.0273797.s011.tif]

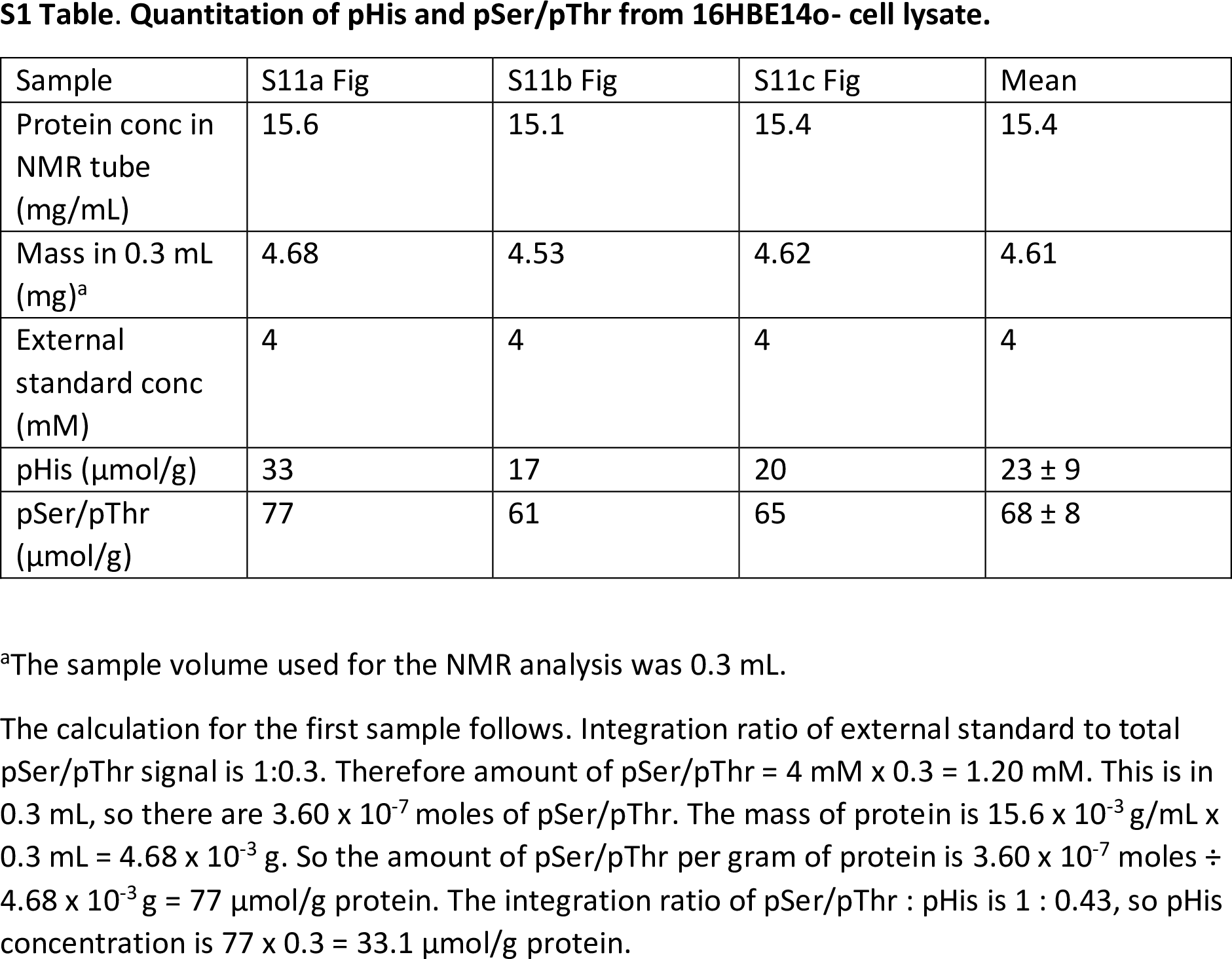

Supplement: S1 Table — (TIF) [file pone.0273797.s012.tif]

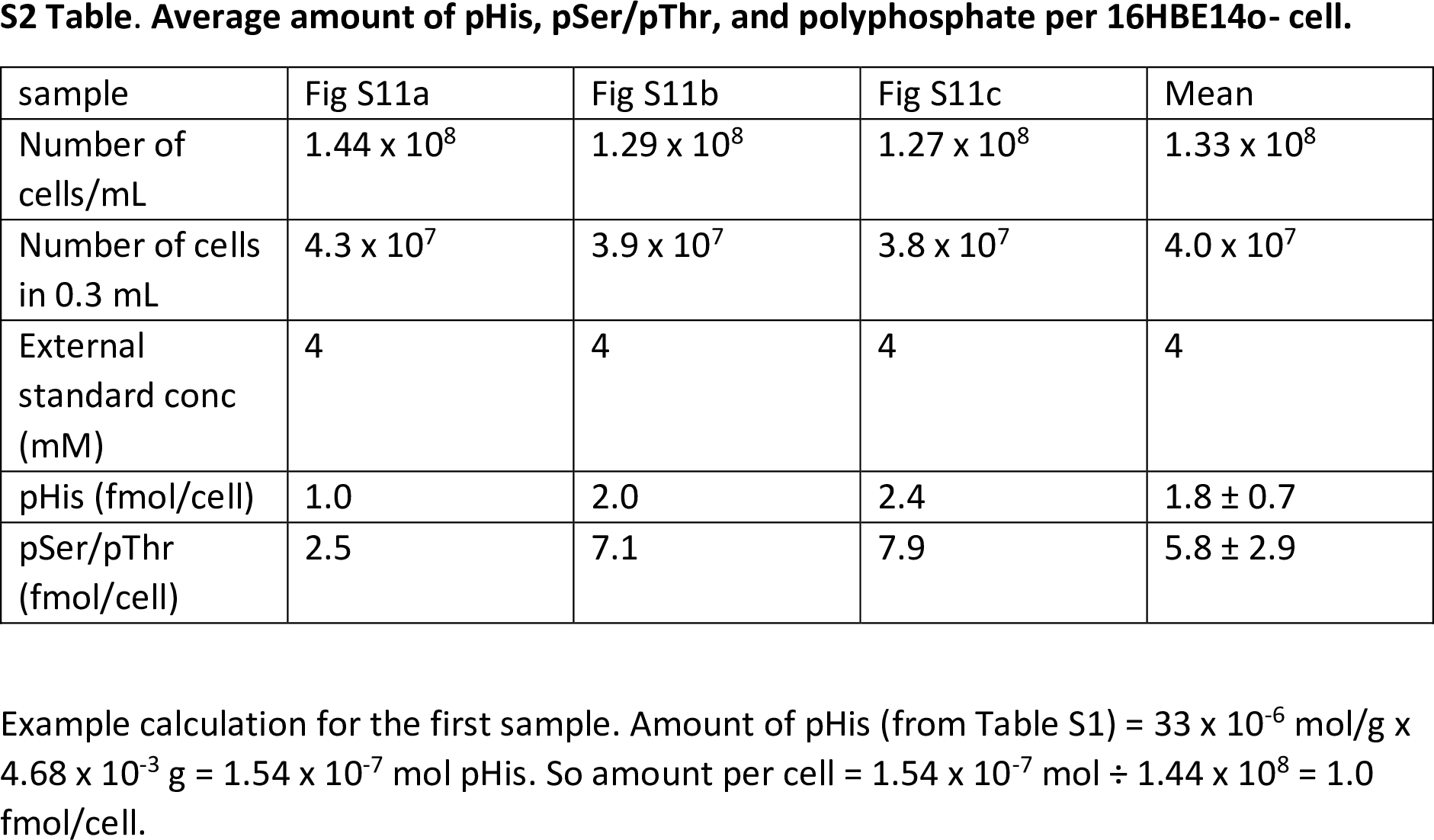

Supplement: S2 Table — (TIF) [file pone.0273797.s013.tif]

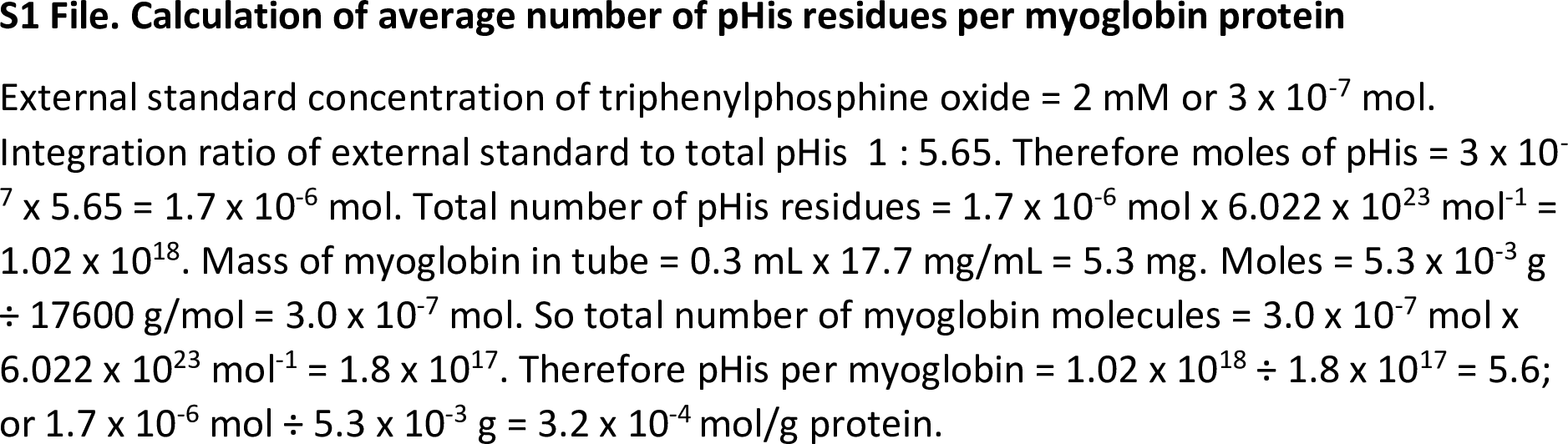

Supplement: S1 File — (TIF) [file pone.0273797.s014.tif]
